# Bistability of Mitochondrial Respiration Underlies Paradoxical Reactive Oxygen Species Generation Induced by Anoxia

**DOI:** 10.1371/journal.pcbi.1000619

**Published:** 2009-12-24

**Authors:** Vitaly A. Selivanov, Tatyana V. Votyakova, Jennifer A. Zeak, Massimo Trucco, Josep Roca, Marta Cascante

**Affiliations:** 1 Departament de Bioquimica i Biologia Molecular, Facultat de Biologia, Institut de Biomedicina at Universitat de Barcelona IBUB and IDIBAPS Hospital Clinic, Barcelona, Catalunya, Spain; 2 Hospital Clínic, IDIBAPS, CIBERES; Universitat de Barcelona, Barcelona, Catalunya, Spain; 3 A.N.Belozersky Institute of Physico-Chemical Biology, Moscow State University, Moscow, Russia; 4 Department of Pediatrics, The University of Pittsburgh School of Medicine and The Children's Hospital of Pittsburgh, Diabetes Institute, Pittsburgh, Pennsylvania, United States of America; Northwestern University, United States of America

## Abstract

Increased production of reactive oxygen species (ROS) in mitochondria underlies major systemic diseases, and this clinical problem stimulates a great scientific interest in the mechanism of ROS generation. However, the mechanism of hypoxia-induced change in ROS production is not fully understood. To mathematically analyze this mechanism in details, taking into consideration all the possible redox states formed in the process of electron transport, even for respiratory complex III, a system of hundreds of differential equations must be constructed. Aimed to facilitate such tasks, we developed a new methodology of modeling, which resides in the automated construction of large sets of differential equations. The detailed modeling of electron transport in mitochondria allowed for the identification of two steady state modes of operation (bistability) of respiratory complex III at the same microenvironmental conditions. Various perturbations could induce the transition of respiratory chain from one steady state to another. While normally complex III is in a low ROS producing mode, temporal anoxia could switch it to a high ROS producing state, which persists after the return to normal oxygen supply. This prediction, which we qualitatively validated experimentally, explains the mechanism of anoxia-induced cell damage. Recognition of bistability of complex III operation may enable novel therapeutic strategies for oxidative stress and our method of modeling could be widely used in systems biology studies.

## Author Summary

The levels of reactive oxygen species (ROS) that are generated as a side product of mitochondrial respiratory electron transport largely define the extent of oxidative stress in living cells. Free radicals formed in electron transport, such as ubisemiquinone, could pass their non-paired electron directly to oxygen, thus producing superoxide radical that gives rise to a variety of ROS. It is well known in clinical practice that upon recommencing oxygen supply after anoxia a tissue produces much more ROS than before the anoxia, and the state of high ROS production is stable. The mechanism of switching from low to high ROS production by temporal anoxia was unknown, in part because of the lack of detailed mathematical description of hundreds of redox states of respiratory complexes, which are formed in the process of electron transport. A new methodology of automated construction of large systems of differential equations allowed us to describe the system in detail and predicts that the mechanism of paradoxical effect of anoxia-reoxygenation could be defined by the properties of complex III of mitochondrial respiratory chain. Our experiments confirmed that the effect of hypoxia-reoxygenation is confined by intramitochondrial processes since it is observed in isolated mitochondria.

## Introduction

The pathologic consequences of anoxia–reoxygenation, including the oxidative stress associated with increased production of reactive oxygen species (ROS) in mitochondria, form the basis of major diseases, including heart disease, age-related degenerative conditions and ischemic syndrome in reperfusion [1]. The use of novel antioxidants, which addresses the consequences of oxidative stress, has proven to be effective in organ preservation [2], but there is no doubt that a better understanding of the causes of elevated ROS production during the anoxia/reoxygenation would help to introduce novel strategies addressing the primary events of this clinical phenomenon.

Paradoxically, ROS production increases during severe hypoxia despite of decrease of oxygen concentration; this ROS increase acts as a metabolic signal for cell adaptation to oxygen deficiency [3]. Moreover, when cells that have been exposed to anoxia are returned to their normal oxygen supply, the rate of ROS production, instead of returning to the low baseline, greatly increases, often leading to cell death. This phenomenon of reperfusion injury after ischemia has been well known [4],[5], but its mechanism remains unclear and it is analyzed here.

**Figure 1:**
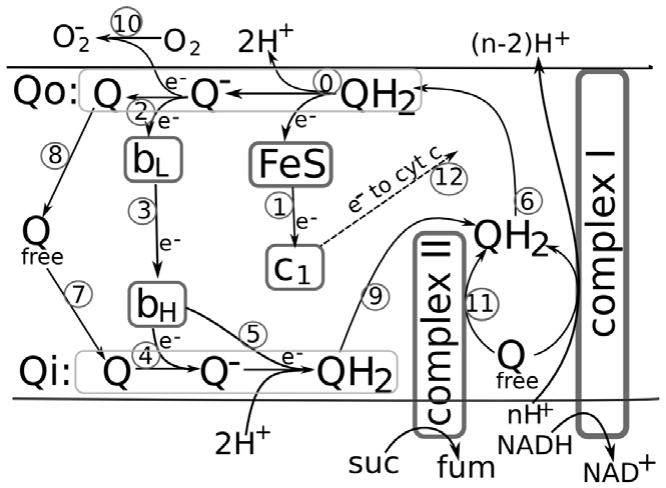
Scheme of the redox reactions in complex III of mitochondrial respiratory chain. Explanation is given in the text.

Mitochondrial respiratory electron transport, which is schematically shown in Figure S1, is generally accepted as a process related with ROS production in living cells. In particular, o-site of quinol oxidation in complex III (Qo) is one of the most frequently considered sites of superoxid anion generation [3],[6],[7], and the considered here mechanism for the stimulation of ROS production by anoxia is related to this site. The proposed mechanism is based on our analysis of the well-known Q-cycle (ubiquinone (Q) oxidation/reduction) mechanism of electron and proton transport performed by complex III in the mitochondrial respiratory chain [8]–[13], as schematically shown in Figure 1. The main components of complex III are cytochrome b, containing two hemes characterized by low (b_L_) and high (b_H_) midpoint potentials, Rieske protein containing an iron-sulfur redox center (FeS), and cytochrome c_1_. The FeS center of the Rieske protein accepts one electron from a bound ubiquinol (QH_2_) producing a highly reactive anion radical of ubiquinone (Q^−^) known also as semiquinone radical (further referred as SQ) and releasing two H^+^ to the cytosolic side. The electron accepted by the FeS center then is delivered to c_1_ and passes further downstream in the electron transport. The semiquinone radical is normally transformed into ubiquinone as it delivers its unpaired electron to cytochrome b_L_, which then passes it to b_H_. However, there is a probability that the semiquinone radical delivers its unpaired electron directly to oxygen, producing a superoxide radical [14]–[16]. Semiquinone oxidation by any of these two mechanisms transforms it into ubiquinone, which then dissociates from complex III, binds at the matrix side and receives two electrons from cytochrome b_h_ that are derived from oxidation of two QH_2_ molecules as described above. This process subsequently generates semiquinone and QH_2_ again, taking protons from the matrix. The dissociation of the newly produced QH_2_ and its subsequent binding at the cytosolic side starts the cycle again.

A great deal of problems in studying ROS production in respiratory chain induced by anoxia arises because of the lack of tools for systematic theoretical analysis of this complicated system. Various combinations of oxidized and reduced states of the electron transporters create 400 possible different redox states for complex III, which are formed in the process of electron transport and which should be taken into account in an analysis of electron transport and the related ROS production. The high levels of complexity has, until now, precluded understanding of complex III functionality. To overcome this problem, we have developed a special software tool that takes into account all possible redox states of complex III. The software automatically constructs a system of differential equations describing their evolution based on several rules defined by the types of reactions between the electron transporters, as outlined in Methods and in more detail in Text S1. Our current approach to simulating mitochondrial respiration has inherited the main principles of automated construction of large equation systems used for stable isotope tracer data analysis [17]–[19].

Using this tool for the simulations of evolution of all redox states of complex III, we found that complex III has a property of bistability: it can persist in two different steady states at the same set of parameters; evolution to one or another steady state is defined only by the initial state of the system. One such state is characterized by a high ROS production rate. The system can be switched to this latter state either by an increase in succinate supply or by a decrease of oxygen availability, and can persist in it after a return to the initial conditions. This behavior explains the mechanics of paradoxical increase in ROS generation induced by anoxia and its further increase after return to a normal oxygen supply. Experimental data presented here qualitatively confirmed such a bistable behavior.

## Results/Discussion

### The phenomenon of bistability in complex III operation

**Figure 2:**
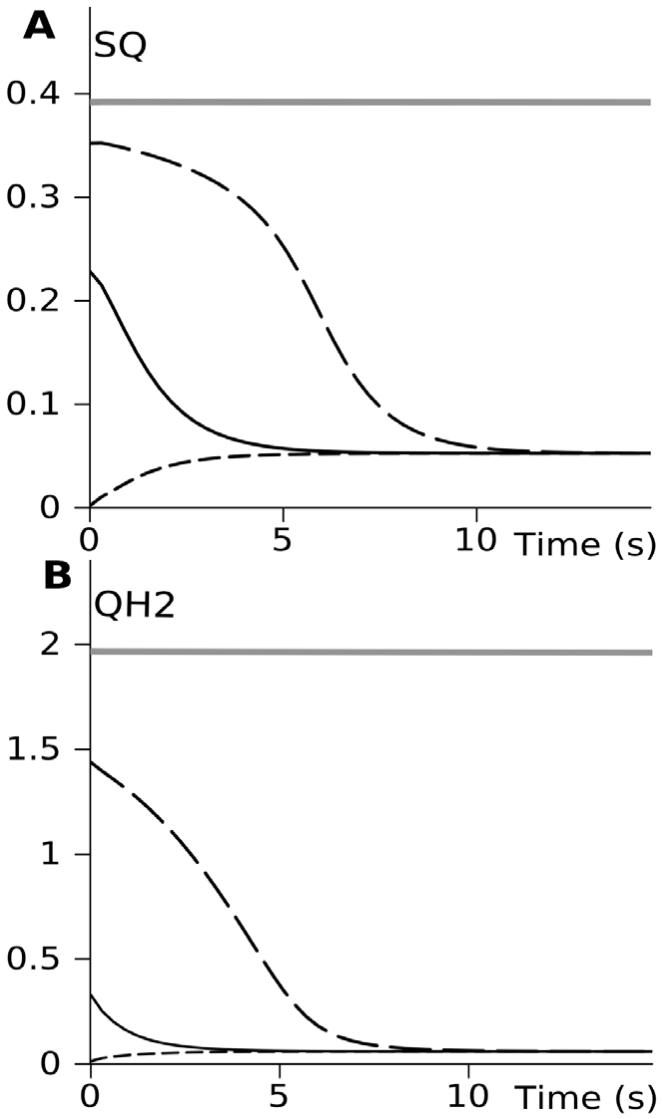
The prediction of bistability in complex III operation. The levels of semiquinone (A) and free ubiquinol (B) were chosen as the indicators of state of system. If the system is in highly reduced state initially, it remains in this state (thick gray curves); if initially the levels of reduction are lower, the system evolves to another steady state characterized by lower levels of semiquinone and, respectively, ROS production. The same type of curves in (A) and (B) refers to the same simulation.

The model constructed, as described in Methods, is based on the generally accepted Q-cycle mechanism of complex III operation and predicts that it can evolve to a one of two different steady states. The direction of complex III evolution depends on the initial state of the system. If initially it is in highly reduced state, characterized by high levels of ubiquinol and semiquinone, it remains in this state, as shows thick gray lines in Figures 2A and 2B. However, if the initial levels of reduction is lower, the system evolves to another steady state, the same for a variety of initial states, as Figures 2A and 2B show. Thus, the Q-cycle mechanism defines bistability of complex III operation: with the same parameters it could function in a mode characterized by either low or high SQ levels (low or high ROS producing states respectively).

### Two branches of steady states in the continuum of succinate concentrations

**Figure 3:**
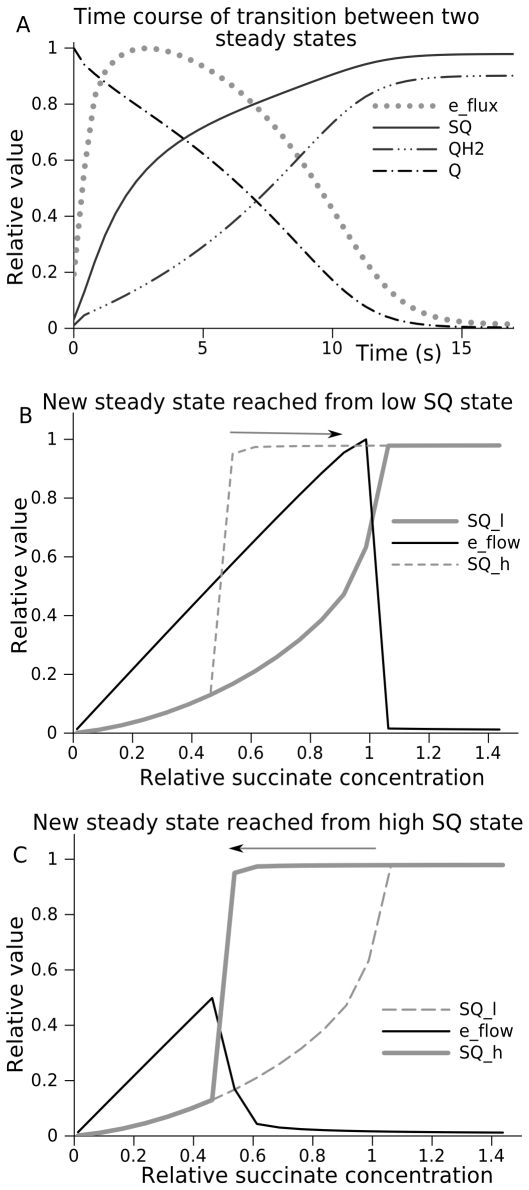
Bi-stability of the reaction system catalyzed by complex III. (A) shows the time course of transition of complex III from low to high ROS producing steady state. This transition was triggered in the model by a switch to higher succinate supply (increase in succinate concentration) and it is characterized by the increase of content of semiquinone radical bound at outer (Q_o_) site of the complex (SQ) and free ubiquinol (QH_2_ free), and decrease ubiquinone content (Q free). Electron transport rate (e_flux) initially increases, but then decreases because the deficit of electron acceptor (Q) impedes the oxidation of cytochrome b_H_. As a consequence, cytochrome b_L_ remains reduced and cannot accept electrons from bound SQ thus increasing SQ content and related ROS production. (B) shows the computed content of semiquinone radicals bound at Qo site of complex III (SQ) and electron flow rates in steady states reached at various succinate supply from an initial state characterized by low SQ content (SQ_l). When the substrate supply overcomes a certain threshold, the system comes to a steady states characterized by the high levels of SQ and ROS production. If the system initially is in one of such high ROS producing states, it evolves to the different steady states after a decrease of succinate supply. Dashed line shows for comparison the continuum of steady states for SQ reached from an initial state characterized by high SQ content (SQ_h). (C) shows the computed content of semiquinone radicals bound at Qo site of complex III (SQ) and electron flow rates in steady states reached at various succinate supply from an initial state characterized by high SQ content (SQ_h). When the substrate supply decreases below a certain threshold, the system comes to a steady states characterized by the low levels of SQ and ROS production. If the system initially is in a one of such low ROS producing states, it evolves to the different steady states after an increase of succinate supply. Dashed line shows for comparison the continuum of steady states for SQ reached from an initial state characterized by low SQ content (SQ_l).

Various factors could trigger complex III from one mode to another, specifically, the availability of succinate could be a triggering factor. Figure 3A shows the time course of the transition from low to high ROS producing steady state, induced by an increase in succinate concentration (eq (17)). The capacity for ROS production is reflected in the concentration of free radicals, which can directly interact with oxygen, such as the semiquinone radical (SQ) bound to the Q_o_ site of complex III [14],[20]. An increase in succinate supply, and respective reduction of free ubiquinone (Q) to ubiquinol (QH_2_), switches the system from low ROS producing state, characterized by low levels of SQ, to the high ROS producing state, characterized by high levels of SQ. In accordance with mass conservation, reduction of Q to QH_2_ results in the deficiency of Q; this decreases electron flow and promotes reduction of cytochrome b. The QH_2_ bound at Q_o_ site can freely deliver its first electron to the cytochrome c_1_, but cannot deliver the second electron to cytochrome b_L_ because latter is already reduced. As a result, highly active SQ radicals are accumulated.

The continuum of steady state SQ levels and electron flow dependent on succinate supply is shown in Figures 3B and 3C. If the system is initially in a low ROS producing state, it switches to the high ROS production when the succinate concentration reaches a certain threshold value as Figure 3B shows. However, if the succinate concentration decreases back when the system initially is in high ROS producing state, it does not follow the same pattern, as Figure 3C shows. This example demonstrates hysteresis in complex III behavior.

The value of transmembrane potential is essential for such hysteretic behavior. As Figure S2 shows, the region of bistability is clearly distinguishable at transmembrane potential of ∼200 mV, which corresponds to the state 4 of mitochondrial respiration. The fall down of transmembrane potential to below 150 mV (as could be when the addition of ADP switches mitochondria to the state 3 of respiration), switches complex III from high to low ROS producing state. At high transmembrane potential the region of bistability persists over a large variation of model parameters as the sensitivity analysis shown in Text S1 and Figures S3 and S4 indicates. A switch from one steady state to another one essentially redistributes the fractions of various redox states of the complex as Figure S5 shows.

 The electron flux in high ROS producing steady state, being restricted by the deficiency of Q, is low; maintaining it requires low succinate supply and this steady state persists even if substrate supply decreases until it falls down below the minimal threshold, as displayed in Figure 3C. The reduction of molecular oxygen by semiquinone radicals transforms them into Q, producing an acceptor able to take electrons from b_h_, and thus to activate the Q-cycle. Thus, high ROS production rate, in a way, is a means to return back to low ROS production. Even a low rate of such an electron leak to oxygen helps the system to revert to low ROS production (see sensitivity analysis presented in Text S1). Normally direct transfer to oxygen insignificantly contributes to the total electron flow, but in the case of extremely high complex II activity, if it reduces all available Q, the electron flow to complex IV would equalize the ROS production rate.

### Experimental confirmation of bistability

**Figure 4:**
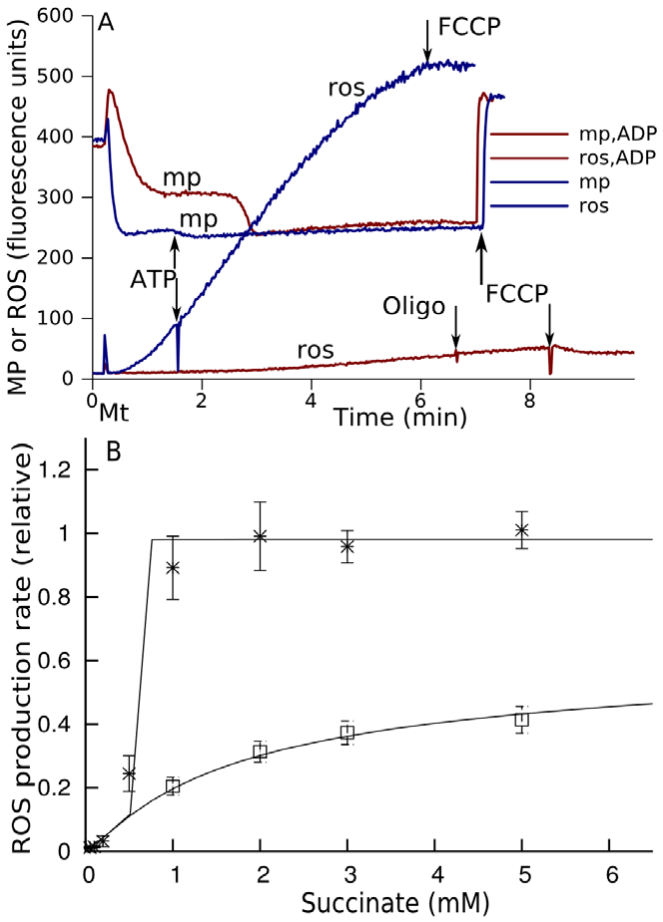
Experimental observations of bistability in mitochondrial electron transport and their simulation in the model. (A) shows the registration of ROS accumulation (traces “ros”) and membrane potential (traces “mp”) and in the suspension of isolated rat brain mitochondria incubated with 5 mM of succinate in the absence (blue traces) and in the presence of 1mM ADP (red traces). In the absence of ADP mitochondria are in high ROS producing state; the presence of ADP switches them to low ROS producing mode. Within the first 3 min initially present ADP is completely converted into ATP; the completion of ATP synthesis could be seen as an increase of membrane potential measured as the quenching of safranin O fluorescence (red trace “mp,ADP”). After the transformation of ADP into ATP and return to the state 4 of respiration, ROS production remained low (red trace “ros,ADP”). Under the conditions where ADP was absent (blue traces), 1 mM of ATP together with 1 uM of oligomycin was added and it did not decrease the rate of ROS production. The reason for addition of oligomycin together with ATP was to avoid effect of small amounts of ADP, which are usually present in preparations of ATP (blue traces). Thus, as the model predicts (Figures 3B and 3C), under the same conditions there exist two different states of ROS production. The complete set of experiments is presented in Text S1. (B) The experiments similar to that shown in (A) performed for various succinate concentrations revealed that two branches of steady state ROS production existed for the same experimental conditions in accordance with the model's prediction (Figures 3B and 3C). Upper branch (asterisks) corresponds to the measured initial rate of ROS production in state 4 (blue curve “ros” in Figure 4A), then 1 mM of ADP was added and, after 3 min of incubation with ADP, oligomycin was added to assure the transition back to state 4 of respiration. The temporal ATP synthesis switched the respiratory chain into low ROS production mode persisted after return to state 4 of respiration. The rate measured in this state (corresponded to the red trace “ros,ADP” in Figure 4A) constituted the lower branch (squares). Lines are the model simulations where the relative succinate concentration is transformed into mM using a scaling factor (the same for all points), chosen so that the threshold for stepwise shift to high ROS production in the upper branch in simulation corresponds to the one obtained experimentally. The succinate dehydrogenase is considered here as a Michaelis' function of not only Q, but also succinate (see eq (17) in “Methods”). Upper steady states were reached in the model when initially the system was in a high ROS producing state, lower steady states were reached when initially the system was in a low ROS producing state.

The predicted phenomenon of bistability explored in Figure 3 could be observed experimentally, as Figure 4A shows. The isolated rat brain mitochondria incubated with succinate (state 4 of respiration) are in a high ROS producing state (blue trace “ros”), however, temporal presence of ADP, which is rapidly transformed into ATP, switches them to a low ROS producing mode (red trace “ros,ADP”). When ADP is present, ATP synthesis lowers membrane potential (shown in reverse direction as measured by quenching of fluorescence, red trace “mp,ADP”). However, after complete transformation of the added ADP into ATP, mitochondria again come back to state 4 of respiration, the membrane potential increases to the same levels as that without ADP (blue trace “mp”), but ROS production remains to be much lower (red trace “ros,ADP”). ADP only switched respiratory chain from high to low ROS production state, which could be maintained under the same conditions of incubation. The addition of ATP to the medium had no effect on ROS production (blue trace “ros”). In this case ROS production was the same as in the absence of ATP (Figure S6).

Figure 4B shows two branches of steady state ROS production rates measured before addition of ADP (stars) and after its consumption (squares) (as Figure 4A explains). Solid lines generated by computer simulation show that our model has reproduced these experimental data after implementing the Michaelis' dependence of complex II activity on succinate concentration (eq (17)). Thus, isolated mitochondria show the predicted bistable behavior (Figure 3) and the model simulates the particular experimental data (Figure 4B). In these experiments 5 mM of pyruvate were present at variable concentrations of succinate to ensure that overall substrate supply is not a limiting factor. In parallel experiments it was shown that at different succinate concentrations membrane potential was invariable (not shown). It should be noted that the parameters used as characteristics of succinate oxidation (Vm, Km) in complex II, in fact characterize several processes including succinate transport. In our *in vitro* experiments the amount of succinate must be sufficiently high to maintain the rate of its transport needed for ubiquinone reduction. The intramitochondrial succinate concentration, which *in vivo* is produced in TCA cycle and which is sufficient to trigger high ROS production, could be much less than that necessary to be added externally. The actual parameters, which govern the oxidation of succinate produced in the matrix *in vivo*, could be different, and since phenomenologically the mechanism admits the forward and reverse switch between the steady states (Figure 3), it could be expected under some conditions, such as ischemia, which could induce several fold increase in intracellular succinate content and, as a consequence, increase in FADH_2_ [21]. Another factor, which substitutes succinate in triggering ROS production *in vivo*, is reduction of ubiquinone by complex I, whose substrate, NADH, is accumulated during anoxia [21].

### Anoxia-induced switch to high ROS producing steady state

**Figure 5:**
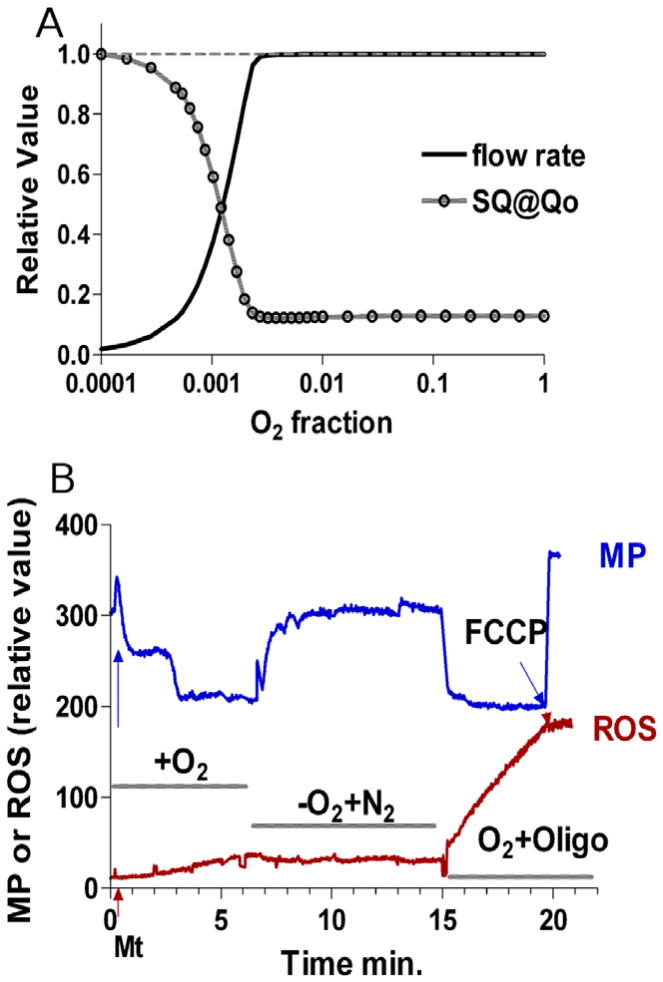
Theoretical predictions and experimental observations of the bistability in mitochondrial electron transport triggered by anoxia. (A) shows that decrease of oxygen concentration down to 0.1% O_2_ and below induces a switch to high SQ content if initially the system is in a state characterized by low SQ content. The dependence of SQ content at Q_o_ site of complex III was calculated using the oxygen dependence of complex IV activity summarized in eq (18) of Model section. The increase of SQ content at Qo site proceeds in conjunction with decrease of electron flow limited by oxygen availability. Dashed line shows the continuum of steady states for SQ content reached if initially mitochondria are in a high ROS producing state. In this case the increase of oxygen availability does not restore low levels of SQ radicals. (B) shows that mitochondria, switched to a low ROS producing state by a temporal presence of ADP (as in (Fig 4A), could be switched back to high ROS production by a short application of anoxia (red trace), which is in accordance with the model prediction shown in Figure 4A. Anoxia was induced by substitution of atmospheric O_2_ for N_2_ and its effectiveness was estimated by the decrease of transmembrane potential (blue trace). After reoxygenation followed by restoration of membrane potential, ROS production is essentially higher than before anoxia.

The lack of oxygen itself can induce a switch to high ROS production if initially respiratory chain is in a low ROS production mode as Figure 4A shows. For this simulation the electron flux from complex III to complex IV was described as Michaelis' function of oxygen concentration (eq (18)). Parameters were chosen so that oxygen availability limits the flux when oxygen concentration falls to about 0.1% O_2_, (in accordance with [13],[22]). The decrease of oxygen availability at the excess of succinate results in a reduction of ubiquinone and cytochrome b, and, finally, an accumulation of SQ radicals. As Figure 5A shows, the accumulation of SQ radicals proceeds in conjunction with limiting the electron flow by the lack of oxygen. Apparently, the physiological response to mild hypoxia, which could be observed at 5% O_2_ [23], is related with another mechanisms.

If after passing through nearly anoxic conditions oxygen availability increases, the system remains in a high ROS producing state. Thus, the functional organization of electron transport in complex III, which has the property of bistability, explains the apparent paradox of increased ROS production induced by anoxia and re-oxygenation.

Figure 5B shows that anoxia induces a switch to a high ROS producing mode in isolated brain mitochondria, which were switched to a low ROS producing mode by temporal presence of ADP. Experiment was started with an open cuvette and normal oxygen supply by an addition of mitochondria to the incubation medium containing 1 mM ADP. Low value of membrane potential is indicative of mitochondria being initially in state 3. Upon conversion of all present ADP into ATP, mitochondria transited into state 4; an increased membrane potential is indicative of that transition. During the first stage of experiment mitochondria released low, but measurable amounts of ROS. An anoxic state was achieved by bubbling of the cuvette with N_2_ for 2–3 min and then closing it with a Teflon lid. Slow depolarization of mitochondria and their persistence in the depolarized state for the duration of closure indicated an achievement of anoxic state. Notably, the ROS signal stayed flat during that stage. At the third stage of the experiment, the resumption of oxygen supply was achieved by opening the cuvette and bubbling it with air. At this stage mitochondria regained membrane potential up to the initial level thus showing their metabolic integrity. Significantly higher than at initial normoxic stage amounts of ROS were released upon the resumption of oxygen supply.

Although the damaging effect of reoxygenation after anoxia is well known in general, presented here experimental data for the first time directly demonstrate anoxia-induced increase in ROS at the levels of isolated mitochondria.

**Figure 6:**
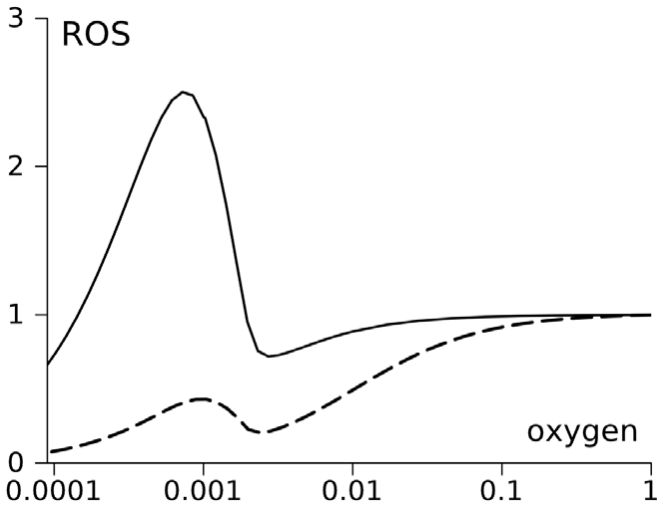
Theoretical predictions of ROS production under hypoxic conditions. The rate of ROS production was calculated using eq. (16) with two different values of K_ROS_ indicated in the Figure. Concentrations of SQ necessary for the calculations were the same as in Figure 5A.

While at constant oxygen concentration the levels of SQ at Qo site could be considered as a relative rate of ROS production, the change of oxygen concentration must also affect it. Figure 6 shows the prediction of ROS production rate when the oxygen concentration changes as Figure 5A shows. For the calculations of ROS production eq. (16) was used with various K_ROS_. Figure 6 indicates that the increase in ROS production could be detectable despite the decrease of oxygen concentration, if K_ROS_ is in the range of Km for respiration. Increase of K_ROS_ results in the decreases ROS production before it starts to affect the respiration rate that induces increase of SQ. In this case the change of SQ would not be well detectable. Thus, if experimentally ROS production was found to increase under anoxic conditions [3], it means that K_ROS_ must be in the range of Km for respiration or lower.

### Conclusions

 The algorithms for automated construction of large systems of differential equations, which are presented in Methods, allowed us to simulate in detail the Q-cycle mechanism of complex III operation and to find that the property of bistability is inherited from this mechanism. Due to this property it can operate in a low or high ROS producing mode at the same values of parameters, which explains the difference in ROS generation by respiratory chain before and after an episode of anoxia. The presented experiments qualitatively confirm the existence of two different ROS producing steady states in isolated mitochondria at the same microenvironmental conditions and a possibility to switch between them by the temporal presence of ADP and/or episodes of anoxia.

The following biochemical events were found to underlie the transition from low to high ROS producing state: (i) ubiquinone pool is reduced to ubiquinol, (ii) lack of oxidized form (ubiquinone) restricts cytochrome b_H_ oxidation and results in b_H_ and b_L_ reduction, which, in turn, (iii) restricts the oxidation of semiquinone radicals, thus increasing their concentration. If substrate concentration is then decreased below a threshold, the remaining electron flow is sufficient to maintain this high ROS producing state and therefore low and high ROS producing states could exist at the same set of parameters.

The increase of ROS production in tissues caused by ischemia-reperfusion is a well known clinical phenomenon; it is of great importance in various systemic diseases and organ transplantation. However, it was not known, which system is responsible for the switch from normally low ROS producing state to high ROS production, and how such a switch proceeds. We predicted and experimentally confirmed that complex III could respond to anoxia-reoxygenation in the same manner as it was observed at tissue levels. This indicates that the revealed mechanism could constitute the main part of tissue response to anoxia-reoxygenation. It does not exclude, though, a possible contribution of other parts of respiratory chain and/or other parts of metabolic and signaling pathways as modifiers of the analyzed phenomenon or even as other individual sources of ROS; rather, our findings open a new direction in investigation of the molecular mechanisms of bistable behavior that underlies the signaling effect of anoxia and oxidative stress caused by reoxygenation. In living cells ischemia combines several factors affecting ROS production, such as reduction of electron transport chain, anoxia, converting ATP into ADP and AMP, and deamination of the latter resulting in decrease of cellular adenilate pool [24]. Various combinations of these factors could create various types of behavior. Understanding their underlying mechanisms will improve the clinical care of diseases related to anoxia and also the quality of organ transplantation.

 The proposed method for kinetic description of multienzyme complexes inherited from the established method of automated equation construction, which we developed for a different area of investigation, namely the analysis of stable isotopic isomers distribution in the intermediates of central carbohydrate metabolism [17]–[19]. Thus, it is not restricted by an area of mitochondrial respiration and could be further modified for applications in different areas, for instant for the analysis of metabolic signalling, which requires a description of multiple states of receptor molecules. Using this method will allow further expanding the model, including in it the other parts of electron transport chain and central metabolism, which provides substrates for mitochondrial respiration, ion transport that defines transmembrane potential, etc. Thus, the proposed method of differential equations construction opens a new direction for most detailed mathematical analysis of complex bioenergetic systems.

## Methods

### Model

The model simulates the reactions performed by complex III, which overall expression is:
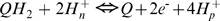
The two electrons taken from QH_2_ then are transferred to cytochrome c and further to complex IV and oxygen in conjunction with translocation of four more protons. Although the presented model does not consider the details of this latter process, it accounts for it stoichiometry.

In the course of complex III reaction its core (consisted of cytochrome b with its two hemes, b_H_ and b_L_, cytochrome c_1_, and Fe-S containing center of Rieske protein) bind and dissosiate ubiquinone/ubiquinol either in the matrix (Qi) or cytosolic (Qo) side of the inner mitochondrial membrane. This binding of quinones produce four different species of the complex:
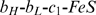
(1)

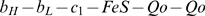
(2)

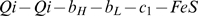
(3)

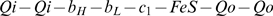
(4)The electron transporters consisting the species (1)–(4) could be either in reduced or oxidized state. Various combinations of redox states of transporters produce many forms of each specie. The concentrations of such forms, which are transformed one into another during the complex operation, are the variables of the model. All the concentrations calculated for different redox forms of the same specie are stored in a specific array. To facilitate the references to variables inside the program, the two redox states of each transporter represented either by the digit “0” for oxidized state or by “1” for the reduced one. For instance, the binary “0011” (or decimal “3”) with respect to the array of core (1) refers to the concentration of a specie with oxidized b_H_ and b_L_ and reduced c_1_ and FeS. On the other hand, these digits together form a binary number, which signifies that the concentration of this redox form occupies third position (starting from 0) in this array. Thus, the program uses such integer number for two purposes: to designate a specific redox form and in the same time to refer to the place of its concentration in the array reserved for the concentrations of all possible redox forms.

The total number of redox forms for a specie is defined by the number of transporters constituting it, being 2^n^, where n is the number of transporters. In this way, core (1) has 16 different redox states varying from 0000 to 1111; species (2) and (3) have 64 states each, varying from 000000 to 111111, and, respectively, the specie (4) has 256 redox states. The program constructs differential equations for each of these redox states based on the algorithms that reflect the redox reactions between transporters in accordance with the known biochemical mechanism of electron transport in complex III. As is schematically shown in Figure 2, the model takes into account 12 types of reactions, nine of them constitute the proper Q-cycle and three others are just related with complex III activity: superoxid anion formation (10), QH_2_ supply by succinate dehydrogenase, and cytocrome c_1_ oxidation by cytochrome c.

The algorithms for automated calculation of reaction rates for each redox form participated in thece 12 types of reactions is described in details next.

Reaction 0 transports the first electron of QH_2_ bound on the positive (p-, or cytosolic) side to Fe3+ of Rieske protein and releases two protons to the intermembrane space.
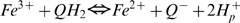
(5)The program simulates this reaction for all redox states of complex III capable to perform it, i.e. for the forms with attached QH_2_ at Qo site and oxidized Fe-S center of Rieske protein. In binary terminology described above for species b_H_-b_L_-c_1_-FeS-Q-Q the reaction (5) could be written as follows:
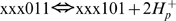
(5a)here x designate any redox state of a respective transporter, 0 or 1. The expressions for reaction rates according to the mass action law are:
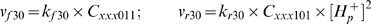
(6)The program passes through the array for species (2), checks if the position of variable satisfy the requirements for substrate given in (5a) (that three last digits are 011), calculates the position of respective product in the array (three last digits are 101 and the digits designated as x are the same as for chosen substrate), then calculates fluxes (6) and adds them to the derivatives for the substrate and product. For the derivatives the program also has an array, where positions are organized in the same way as for concentrations.

For the other species capable to perform this reaction, Qi-Qi-b_H_-b_L_-c_1_-FeS-Qo-Qo, it could be written as
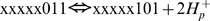
and reaction rates expressed similar to (6).

The model takes into account the ratio of forward and reverse rate constants, which could be defined from the difference of midpoint redox potentials (E_m_):
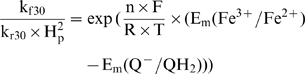
(6a)where n is the number of electrons transported, F = 96500 c/mol is the Faraday constant, R = 8.3 J/(mol×K) is the gas constant, T = 298 K is temperature. The derivation of this equation is based on the basic thermodynamic principles as is described in details in Text S1. The reported values for E_m_ at pH 7 vary as much as 312 mV, [25] or 280 mV, [26] for Fe^3+^/Fe^2+^ and 200 mV, [27] or 300 mV, [28] for Q-/QH2; this variability is reflected in the variability of value expressed by (6a) from 0.6 to 18.

Reaction 1 transports the electron received by Fe^2+^ of Rieske protein from QH_2_ further to c_1_:
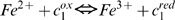
All the species (1)–(4) with reduced FeS center of Rieske protein can perform this reaction:
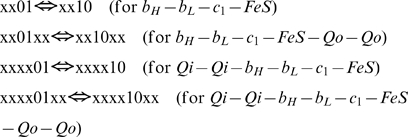
Reaction rates for core species:
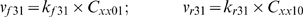
(7)The ratio of forward and reverse rate constants is defined by an equation similar to (6a):
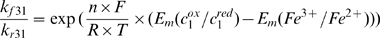
(7a)Midpoint potentials, for Fe^3+^/Fe^2+^ of Riske protein is 312 mV [25] and for c_1_ it is 341 mV [29]. This difference (ΔE_m_ = 29 mV) results in three-time difference in forward and reverse rate constants.

Reaction 2 transports the second electron of Qo to b_L_:
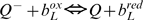
Only the species with ocupied Qo site perform this reaction:
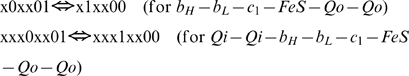
Reaction rates for b_H_-b_L_-c_1_-FeS-Qo-Qo:
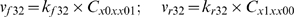
(8)The reported E_m_ (−60 mV for b_L_ [30] and −140 mV for Q/Q^−^ [28]) giving ΔE_m_ = 80 mV according to the equation similar to (7a) define the ratio of forward to reverse rate constant as 25.

Reaction 3 transports electron from b_L_ to b_H_

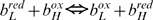
All species with reduced b_L_ and oxidized b_H_ perform this reaction:
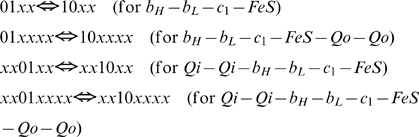
Reaction rates for core species:
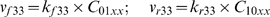
(9)Since in this reaction electron moves in the direction from positive outer side to negative matrix side of the membrane, the rate constants depend of electric transmembrane potential [31].
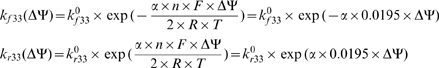
where ΔΨ is transmembrane potential.

Here the ratio of forward and reverse k^0^ is defined by midpoint redox potential difference (similar to (7a)). The reported values are −58 mV for b_L_ [30] and 61 mV for b_H_ [30]. This difference (ΔE_m_ = 119 mV) defines the forward rate constant to be two orders of magnitude higher than the reverse rate constant.

Reaction 4 transports first electron from b_H_ to Q on the n-side:
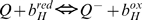
This reaction is performed by the species with ubiquinone bound at Qi site:
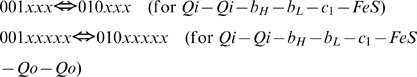
Reaction rates:
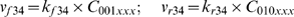
(10)For b_H_ we accepted E_m_ = 61 mV, reported in [30]; for Q/Q^−^, the reported E_m_ vary from 90 mV [30] to 45 mV [32]. To start, we accepted the value which is close to E_m_ for b_H_, in this case the forward and reverse rate constants are equal.

Reaction 5 transports second electron from b_H_ to Q^−^ on the n-side:
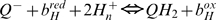
This reaction can be performed by species that contain semiquinone bound on Qi site:
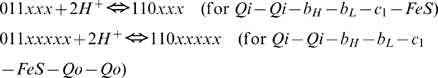
Reaction rates:
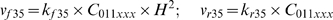
(11)For b_H_ E_m_ = 61 mV [30]; for Q^−^/QH_2_ data in literature vary from 16.5 mV [30] to 150 mV [32]. To start, for this transition we also accepted the value close to E_m_ for b_H_, in this case the forward and reverse rate constants are equal.

Reaction 6. Ubiquinol binding to and dissociation from the complex III at the cytosolic side (Qo) of the inner mitochondrial membrane. This is a way of transition between species: core (1) and species with occupied Qo site (2), and also (3) and (4):
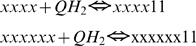
(12)The expressions for forward and reverse reaction rates are:
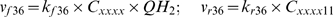
(12a)Simulating this and other binding/dissociation reactions the program works with two arrays, since substrate and product are belong to different species. This does not change much the principles of automated calculation described for the reaction 0. In the same way the program checks the requirements for substrate position (in this case all redox form satisfy it) calculates the position of product specific for each redox form of substrate, calculates the rates (12a) and adds them to the respective derivatives.

For the reaction (12)
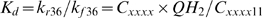
As a starting point we assumed that C_xxxx_ = C_xxxx11_ when QH_2_ = Q. In this case K_d_ = 2 nmol/mg of protein.

Reaction 7. Ubiquinone binding and dissociation to the complex III at the matrix side of the inner mitochondrial membrane:
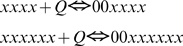
(13)The expressions for reaction rates:
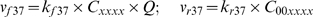
(13a)In simulations we used the same value:
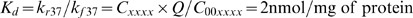
Reaction 8. Ubiquinone dissociation and binding to the complex III at the cytosolic side of the inner mitochondrial membrane:
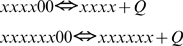
The expressions for reaction rates:
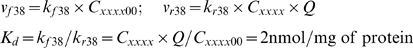
(14)Reaction 9. Ubiquinol dissociation and binding to the complex III at the matrix side of the inner mitochondrial membrane:
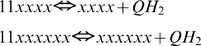
The expressions for reaction rates:
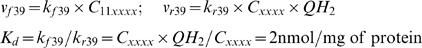
(15)Reaction 10. ROS production in the model.

Semiquinone bound to the Qo site of complex III could be oxidized by molecular oxygen, thus producing superoxide radical:
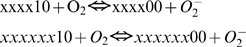
This reaction is considered as unidirectional:
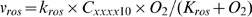
(16)For relative ROS production k_ROS_ was taken to be 1 and K_ROS_ is specified in the legend to Figure 6.

Reaction 11. Succinate oxidation in complex II resulted in ubiquinone reduction (Q to QH_2_), which rate V is described by the Michaelis' equation:
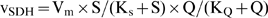
(17)where K_Q_ = 0.5 nmol/mg of protein, K_S_ = 0.25 mM of protein, V_m_ = 1.88 nmol/mg of protein/s, and S is external succinate concentration. Thus, equation (17) represents succinate dehydrogenase activity in a generalized form not going into details as in above reactions.

Reaction 12. Electron transport from complex III to cytochrome c and further to complex IV.

All species (1)–(4) contribute to this rection:
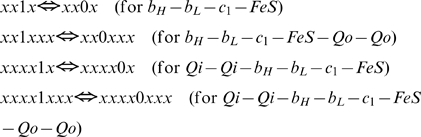
The expression for this reaction rate, which in fact integrates all the steps of electron transition from cytochrome c1 to oxygen, takes into account oxygen concentration:
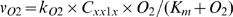
(18)here O_2_ is a fraction of dissolved oxygen with respect to its content at equilibrium with atmospheric oxygen at normal pressure, k is reaction rate constant, K_m_ is Michaelis constant for interaction with oxygen. These parameters were adjusted so that the model describes limitation of electron flow by oxygen, which was found experimentally. According to the experimental data [22],[13], the electron flow is not limited by O_2_ availability until the oxygen concentration falls to about 1 µM (<0.1% O2). The model reproduces these data if K_m_ = 0.03, and k = 25 s^−1^. These parameters were used to calculate the oxygen dependence of semiquinone radical content presented in Figure 5A of the main text.

Complex III content is 0.4 nmol/mg of protein [33], and total ubiquinone content is 4 nmol/mg of protein [33].

### Automated construction of differential equations

The system of differential equations describes the evolution of concentrations (C) of all redox forms, which the program references as is described above. Derivative of each redox state is calculated as a sum of all the reaction rates of influx to or efflux from the considered state, which are calculated in accordance with the equations (6–18). For each type of reaction there is a special function, which, once called for a specie, passes from redox state 0 to the last one checking if the redox conditions permitting the reaction are satisfied and, if yes, calculates the flux and adds it (with respective sign) to the derivatives of concentrations of reagents. In this way, to simulate the operation of respiratory complex it is necessary just to check whether the functions simulating all the reactions performed by the species (1)–(4) are called, and these functions automatically would calculate the derivatives for all 400 redox states of complex III.

For the numerical solutions of differential equations the program implements 5th order Runge-Kutta, Bulirch-Stoer, implicit Bulirch-Stoer, Rosenberg methods [34]. The program and brief user guide is free available in http://www.bq.ub.es/bioqint/selivanov.htm

### Experimental procedures

Similar technique is described in more detail elsewhere [35],[36].

Isolation of rat brain mitochondria - All procedures involving animals were approved by the Children's Hospital of Pittsburgh and were in compliance with “Principles of Laboratory Animal Care” and the current laws of the United States. Rat brain mitochondria were isolated from the cortex of adult Wistar rats. After removal, tissue was minced and homogenized in ice-cold isolation buffer I (IB I) which contained: 225 mM mannitol, 75 mM sucrose, 5 mM HEPES buffer (pH adjusted to 7.3 with KOH), 0.1 mg/ml fatty acid free BSA, 1 mM tetrapotassium EDTA and 12% Percoll. The homogenate thus obtained was carefully layered on the top of a discontinuous gradient of Percoll (24% and 42%) prepared using the same buffer. The preparation was then centrifuged at 31,000×*g* for 10 min. The fraction containing the mitochondria located between 42% and 24% Percoll was carefully withdrawn by a syringe and washed from Percoll twice by pelleting in IB I. The resulting mitochondrial suspension was diluted in isolation buffer II (IB II), which was same as IB I, except for the concentration of EDTA (0.1 mM) and lack of albumin, and spun down at 12,000×*g* for 10 min. The deposit of mitochondria was homogenized in IB II at a final protein concentration of ∼20 mg/ml and stored on ice until use. The protein concentration in the mitochondrial samples was determined using a Protein Assay kit (Pierce Chemical Company, Rockford IL) according to the manufacture's instructions. Mitochondria prepared in this way were active for at least 4–5 hours, as determined by their ability to maintain a stable transmembrane potential in the presence of oxidizable substrates.

Hydrogen peroxide measurements were performed in a stirred cuvette mounted in a Shimatzu RF-5301 spectrofluorimeter maintained at 37°C. Mitochondria (0.2 mg/ml of protein) were added to the incubation medium that contained: 125 mM KCl; 2 mM KH_2_ PO_4_; 2 mM MgCl_2_; 10 mM Tris;10 mM HEPES (pH 7.0); 100 µM EGTA; 5 mM pyruvate and particular concentrations of succinate (0.05–5 mM) as the oxidizable substrates. Reporting fluorescent dye for H_2_O_2_ was Amplex red (2 µM) which increased fluorescence upon oxidation to resorufin in the presence of 1 U/ml of horseradish peroxidase (HRP) as previously described [36]. Measurements were carried out at excitation/emission wavelengths of 560 nm (slit 1.5 nm)/590 nm (slit 3nm), respectively. Amounts of H_2_O_2_ released by mitochondria were estimated by constructing calibration curves using known H_2_O_2_ concentrations in the standard incubation buffer together with Amplex red and HRP, but without mitochondria. Normoxia experiments were performed in a conventional square (10×10 mm), open cuvette. Anoxic experiments were performed in a cuvette with a narrow rectangular neck (10×3 mM). Anoxic conditions were made by bubbling the cuvette with N_2_ and, once the reaction media was depleted of O_2_, closing it with a Teflon lid. Resumption of oxygen supply was achieved by opening the cuvette and bubbling it with air.

Mitochondrial transmembrane potential, ΔΨ_m_ - was estimated using fluorescence quenching of the cationic dye safranine O. Since polarized mitochondria have a negative charge inside, positively charged molecules of safranine O are accumulated inside the matrix; increase in dye concentration inside the matrix leads to fluorescence quenching , thus a decrease in fluorescence corresponds to an increase of membrane potential. The excitation wavelength was 495 nm (slit 3nm) and emission 586 nm (slit 5nm), and the dye concentration used was 2.5 µM [36].

### Ethics Statement

All procedures involving animals were approved by the Children's Hospital of Pittsburgh and were in compliance with “Principles of Laboratory Animal Care” and the current laws of the United States.

## Supporting Information

Text S1 Text file describing the algorithms, parameters, experiments referred in the main text of the manuscript
(0.16 MB PDF)

Figure S1 Scheme of the processes in mitochondrial electron transport chain and their coupling with central energetic metabolism. Respiratory complexes (CI–CIV) deliver electrons from NADH oxidizing it to NAD+ to oxygen reducing it to H2O. This delivery is subdivided into many steps, some of which are coupled with proton transport from matrix to cytosol. Redox reactions in electron transport chain and proton translocation are fed by glycolysis and TCA cycle, which provide NADH, complex 1 substrate, and succinate, complex II substrate. Ubiquinone/ubiquinol (Q/QH2) cycle plays essential role in proton transport and communication between CI–CIII. The scheme of electron transport coupled with proton translocation in the complex III (Q-cycle mechanism) is shown in more detail in the right panel. Oxidizes ubiquinol reducing cytochrome C and releasing two protons to the cytosolic side (positive or p-side; this is reflected in the index of released H+). In addition it translocates two protons from matrix (negative or n-side) to cytosol. Ubiquinol (QH2) delivers its first electron to Fe(3+), releasing 2 protons at the p-side of the inner mitochondrial membrane and producing semiquinone (SQ) radical. Then the latter gives its unpaired electron to cytochrome bl and produced ubiquinone (Q) dissociates from the complex. Free Q is bound at the n-side, receives two electrons from cytochrome bh resulted from oxidation of two QH2 molecules, thus producing subsequently SQ and QH2, taking protons from n-side. Dissociation of the produced QH2 accomplishes a round of the cycle.
(0.06 MB TIF)

Figure S2 Validation of the model by qualitative simulation of the observed experimentally decrease of ROS production and increase of maximal flow rate induced by ADP, and increase of ROS production induced by hypoxia. (a) Decrease of steady state content of semiquinone bound at Qo site (ROS production rate) in conjunction with a decrease of transmembrane potential. Blue squares indicate the steady state levels of SQ bound at Qo site reached from initial states with high SQ levels at various relative succinate concentrations. Orange diamonds indicate the steady state SQ at Qo levels reached from initial states with low SQ levels. These two curves, which indicate bistable behavior of complex III operation, were obtained for transmembrane potential 200 mV that simulates state 4 of respiration. If transmembrane potential falls down to below 150 mV (as could be after a transition to state 3 induced by addition of ADP that stimulates ATP synthesis), the area of bistability is shifted to the right (as indicated by yellow and green triangles) and the same substrate supply corresponds now to low semiquinone content. Thus, if the system operates in high ROS producing mode at the relative succinate levels around 0.8–0.9, addition of ADP would switch it to the low ROS producing mode. (b) Increase of electron flow through the respiratory chain in conjunction with a decrease of transmembrane potential. The labels of curves correspond to the same conditions as in Figure S2a. If the system persists in state 4 of respiration in a high ROS producing mode (orange diamonds), the addition of ADP (transition to state 3) would switch the electron flow to the values indicated by green triangles, and if the substrate supply is around 0.8–0.9, electron flow would increase from 0.4 to 1.4 ng.atom of O/mg/s. (c) Initially mitochondria incubated with succinate are in state 4 and in high ROS production state (orange curve in Figure S2a). The addition of ADP, simulated as decrease of transmembrane potential, induces transition to low ROS production state. After ADP is consumed transmembrane potential increased and ROS production changes to the state indicated by blue curve in Figure S2a. Hypoxia, simulated as further increase of substrate supply and decrease of rate constant of cytochrome c1 oxidation, provoked switch to back to the state indicated by orange curve.
(0.08 MB TIF)

Figure S3 Analysis of sensitivity of the bistability region to the change of model parameters. The basic model parameters, which are listed above and in the main text. To show the sensitivity of bistability region the parameters were changed, which induced the change of area of bistability. Blue and orange lines indicate the bistable region, which corresponds to the indicated above set of parameters. Yellow and green lines illustrate how this region changes in response to the change of a parameter as indicated in the figures.
(0.11 MB TIF)

Figure S4 Analysis of sensitivity of the bistability region to the change of model parameters. Designations are the same as in Figure S3
(0.12 MB TIF)

Figure S5 Distribution of redox states of complex III, which corresponds to low (upper panel), high (lower panel) ROS production states or intermediate non-steady state reached in 3 second after the switch of substrate supply which induced the transition from low to high ROS producing steady state (middle panel). The redox states are positioned as follows: the numbers 0–15 correspond to 16 states of bh-bl-c1-FeS, numbers 16–79 correspond to 64 states of bh-bl-c1-FeS-Q-Q, numbers 80–143 correspond to 64 states of Q-Q-bh-bl-c1-FeS, numbers 144–399 correspond to 256 states of Q-Q-bh-bl-c1-FeS-Q-Q.
(0.08 MB TIF)

Figure S6 (a) Temporary stimulation by ADP switches the electron transport from high to low ROS production mode. Rat brain mitochondria were added to the incubation medium, with subsequent addition of 1 mM ADP (red curves), or with 1 mM of ADP present initially (blue curves). Without ADP, ROS production is much higher than in its presence, however, after ADP addition it decreases. During the transformation of ADP into ATP the membrane potential remains low but, when all added ADP is transformed, the potential increases (curves MP). However, after the completion of transformation, ROS production increased, but not to the original level. Since the produced ATP itself does not affect ROS production, this data indicate that ADP plays a role of trigger, which switches the respiratory chain from high to low ROS production. (b) ATP does not affect ROS production, but adventitious ADP in the ATP preparation was sufficient to switch the electron transport to low ROS producing mode. Blue curves are shown for comparison; they are taken from an experiment, similar to that shown in (a). Black curve shows that ATP added with oligomycin does not change ROS production. Green curves show that a presence of traces of ADP in the preparation of ATP switches a part of mitochondria to low ROS producing mode, and that the addition of oligomycin after ATP does not bring the ROS rate to the original level.
(0.05 MB TIF)
