# Supplementary material for: Bistability of Mitochondrial Respiration Underlies Paradoxical Reactive Oxygen Species Generation Induced by Anoxia
Source: PLoS Comput Biol. 2009 Dec 24;5(12):e1000619. doi: 10.1371/journal.pcbi.1000619 (PMC2789320; doi:10.1371/journal.pcbi.1000619)
Supplement: Text S1 — Text file describing the algorithms, parameters, experiments referred in the main text of the manuscript (0.16 MB PDF) [file pcbi.1000619.s001.pdf]

The ratio of forward and reverse rate constants in the redox reactions of respiratory complex III, as evaluated from the midpoint redox potentials of transporters.

The place of complex III in mitochondrial respiratory chain is shown in Figure S1. It performs the function of proton translocation using the energy of oxidation of ubiquinol that provides succinate dehydrogenase (complex II) and complex I. The oxidation of ubiquinol in complex III results in reduction of cytochrome c, which is oxidized in complex IV. The redox reactions performed by complex III is shown in more detail schematically in Figure 1 of the main text and described next.

Reaction 0. Transport of the first electron from QH_2 bound on the p-side to Fe3+ of Rieske protein and release of two protons.

$$Fe^{3+} + QH_2 \leftrightarrow Fe^{2+} + Q^{-} + 2H^{+}_{p} \qquad (1)$$

The forward and reverse rate constants could be expressed through midpoint redox potentials and metabolite concentrations using the expressions for redox potential of transporter (tr) at the given pH of the medium ($E_m^{tr,pH}$). Specifically, for semiquinone (Q^-) / ubiquinol (QH_2) redox couple it is written as follows:

$$E(Q^-/QH_2)=E_m(Q^-/QH_2) + RT/(nF)\ \ln(Q^-/QH_2)$$

where Em is midpoint redox potential. For the other component of this reaction the expression is:

$$E(Fe^{3+}/Fe^{2+})=E_m(Fe^{3+}/Fe^{2+}) + RT/(nF)\ \ln(Fe^{3+}/Fe^{2+});$$

At equilibrium the redox potentials are equal ($E(Fe^{3+}/Fe^{2+}) = E(Q^-/QH_2)$):

$$E_m(Q^-/QH_2) + RT/(nF)\ \ln(Q^-/QH_2)=E_m(Fe^{3+}/Fe^{2+}) + RT/(nF)\ \ln(Fe^{3+}/Fe^{2+}); \qquad (2)$$

After simple rearrangement of the terms in (2):

$$E_m(Fe^{3+}/Fe^{2+}) - E_m(Q^-/QH_2)= RT/(nF)\ \ln(Q^-/QH_2) - RT/(nF)\ \ln(Fe^{3+}/Fe^{2+});$$

$$E_m(Fe^{3+}/Fe^{2+}) - E_m(Q^-/QH_2)= RT/(nF)\ \ln(Q^-\cdot Fe^{2+}/(QH_2\cdot Fe^{3+})) \qquad (3)$$

Since $Keq=k_f/k_r =(Fe^{2+}\cdot Q^-\cdot H^2_p)/(Fe^{3+}\cdot QH_2)$, the expression under logarithm in (3) equals to $k_f/(k_r \cdot H^2_p)$:

$$E_m(Fe^{3+}/Fe^{2+}) - E_m(Q^-/QH_2)= RT/(nF)\ \ln(k_f/(k_r \cdot H^2_p))$$

$k_f/(k_r \cdot H^2_p) = \exp((nF/RT)\cdot(E_m(Fe^{3+}/Fe^{2+}) - E_m(Q^-/QH_2)))$

Here and further we omitted the sign (+) for H^+ in equations. Usually midpoint potentials are given for pH=7 or H=10^{-4} mM.

Values of the constants:

F(q/mol)	T (K)	R (J/mol/K)	F/RT (q/J or V^{-1})	F/RT (mV^{-1})	RT/F (mV)
96500	298	8.3	39.0151	0.03902	0.02563

Thus the values of midpoint potentials allows defining equilibrium constant or ratio of forward and reverse constants.

Parameters, their values and references:

Fe^{3+}/Fe^{2+}	280	Em, mV	Trumpower, 1981
Fe^{3+}/Fe^{2+}	312	Em, mV	Link et al,1992
Q^-/QH_2 at Qo	200	Em, mV	Roginsky et al, 1999
Q^-/QH_2 at Qo	300	Em, mV	Mulkidjanyan, 2005; Snyder, 2000
ΔEm	50	mV	
$Keq=k_f/k_r =(Fe^{2+}\cdot Q^-\cdot H^2_p)/(Fe^{3+}\cdot QH_2)$			
$k_f/(k_r\cdot H^2)$	7		

Reaction 1. Transport of the electron received by FeS from QH_2 further to c_1:

$Fe^{2+} + c_1^{ox} \leftrightarrow Fe^{3+} + c_1^{red}$

As above, we consider equilibrium, where the redox potentials for cytochrome c1 and iron-containing Rieske center are equal ($E(c_1^{ox}/c_1^{red}) - E(Fe^{3+}/Fe^{2+})$):

$E_m(c_1^{ox}/c_1^{red})+ RT/(nF) \ln(c_1^{ox}/c_1^{red})=E_m(Fe^{3+}/Fe^{2+})+ RT/(nF) \ln(Fe^{3+}/Fe^{2+})$; (4)

After simple rearrangement of the terms in (4):

$E_m(c_1^{ox}/c_1^{red}) - Em(Fe^{3+}/Fe^{2+}) = RT/(nF) \ln(Fe^{3+}/Fe^{2+}) - RT/(nF) \ln(c_1^{ox}/c_1^{red})$;

$E_m(c_1^{ox}/c_1^{red}) - E_m(Fe^{3+}/Fe^{2+}) = RT/(nF) \ln(c_1^{red}\cdot Fe^{3+}/(c_1^{ox}\cdot Fe^{2+}))$ (5)

Since $Keq=k_f/k_r =c_1^{red}\cdot Fe^{3+}/(c_1^{ox}\cdot Fe^{2+})$, the expression under logarithm in (5) equals to k_f/k_r :

$E_m(c_1^{ox}/c_1^{red}) - E_m(Fe^{3+}/Fe^{2+}) = RT/(nF)\ln(k_f/k_r)$

$k_f/k_r = \exp((nF/RT)\cdot(E_m(c_1^{ox}/c_1^{red}) - E_m(Fe^{3+}/Fe^{2+})))$

Parameters, their values and references:

c_1^{ox}/c_1^{red}	341	Em, mV	Leguijt et al, 1993
Fe^{3+}/Fe^{2+}	280	Em, mV	Trumpower, 1981
Fe^{3+}/Fe^{2+}	312	Em, mV	Link et al,1992
ΔEm	29	mV	

$Keq=k_f/k_r =(Fe^{3+}\cdot c1red)/(Fe2^{+}\cdot c1ox)$

k_f/k_r	3.1001

Reaction 2. Transport of the second electron from Q_p to b_L: $Q^- + b_L^{ox} \leftrightarrow b_L^{red} + Q$

At equilibrium the redox potentials for (Q/Q^-) and (b_l^{ox}/b_l^{red}) are equal:

$E_m(b_l^{ox}/b_l^{red}) + RT/(nF)\ln(b_l^{ox}/b_l^{red}) = E_m(Q/Q^-) + RT/(nF)\ln(Q/Q^-)$; (6)

After simple rearrangement of the terms in (6):

$E_m(b_l^{ox}/b_l^{red}) - E_m(Q/Q^-) = RT/(nF)\ln(Q/Q^-) - RT/(nF)\ln(b_l^{ox}/b_l^{red})$;

$E_m(b_l^{ox}/b_l^{red}) - E_m(Q/Q^-) = RT/(nF)\ln(b_l^{red}\cdot Q/(b_l^{ox}\cdot Q^-))$ (7)

Since $Keq=k_f/k_r = b_l^{red}\cdot Q/(b_l^{ox}\cdot Q^-)$, the expression under logarithm in (7) equals to k_f/k_r:

$E_m(b_l^{ox}/b_l^{red}) - E_m(Q/Q^-) = RT/(nF)\ln(k_f/k_r)$

$k_f/k_r = \exp((nF/RT)\cdot(E_m(b_l^{ox}/b_l^{red}) - E_m(Q/Q^-)))$

Parameters, their values and references:

Q/Q- at Qo	-140	Em, mV	Snyder, 2000
Q/Q- at Qo	-150	Em, mV	Roginsky et al, 1999; Mulkidjanyan, 2005
b_l^{ox}/b_l^{red}	-58	Em, mV	Covian et al, 2007
b_l^{ox}/b_l^{red}	-142	Em, mV	Leguijt et al, 1993
ΔEm	82	mV	

$Keq=kf/kr =(Q-\cdot bl^{red})/(Q\cdot bl^{ox})$

k_f/k_r	24.5

Thus, the transfer of first electron from QH_2 to FeS protein is characterized by the change in E_m of ~140 mV,

and the transfer of second electron is related with opposite change of E_m of ~-220 mV. However, the reactions of $Q/Q^{\cdot-}$ and $Q^{\cdot-}/QH_2$ must be coupled so that the exergonic transfer of an electron to cytochrome FeS center provides the energy for reduction of the low potential b_l (Trumpower, 1981a).

Reaction 3. *Transport from b_l to b_h ($b_l^{red} + b_h^{ox} \leftrightarrow b_h^{red} + b_l^{ox}$)*

In this transition electron covers essential distance against the ΔΨ, therefore the equilibrium constant K_{eq} depends on ΔΨ.

$K_{eq}(\Delta\Psi) = K_{eq0}\cdot\exp(-\alpha\cdot n\cdot F\cdot\Delta\Psi/(RT)) = K_{eq0}\cdot\exp(-\ \alpha\cdot 0.039\cdot\Delta\Psi)$, where α is a part of mitochondrial membrane thickness which this electron transition covers and K_{eq0} is the equilibrium constant at □□ = 0. If the mitochondrial membrane thickness is ~6 nm (Rich 2003), and the distance between the two cytochrome b hemes is 2 nm, (Yu 1999) then α= 0.33.

If the electric field affects the forward and reverse rate constant by the same value (but different sign), then they depend on the transmembrane potential as follows (Reynolds 1985):

$k_f(\Delta\Psi) = k_{f0}\ \exp(-\alpha\cdot n\cdot F\cdot\Delta\Psi/(2RT)) = k_{f0}\cdot\exp(-\ \alpha\cdot 0.0195\cdot\Delta\Psi)$,

$k_r(\Delta\Psi) = k_{r0}\ \exp(\alpha\cdot n\cdot F\cdot\Delta\Psi/(2RT)) = k_{r0}\cdot\exp(\alpha\cdot 0.0195\cdot\Delta\Psi)$.

Specifically, $k_f(\Delta\Psi) = 350\cdot k_{r0}\ \exp(-\ 0.0065\ \Delta\Psi)$;

$k_r(\Delta\Psi) = k_{r0}0\ \exp(0.0065\ \Delta\Psi)$;

k_{f0} and k_{r0} are defined by midpoint potentials in the way similar to that shown above.

At equilibrium the redox potentials for (b_h^{ox}/b_h^{red}) and (b_l^{ox}/b_l^{red}) are equal:

$$E_m(b_h^{ox}/b_h^{red}) + RT/(nF)\ \ln(b_h^{ox}/b_h^{red}) = E_m(b_l^{ox}/b_l^{red}) + RT/(nF)\ \ln(b_l^{ox}/b_l^{red}); \quad (8)$$

After simple rearrangement of the terms in (8):

$$E_m(b_h^{ox}/b_h^{red}) - E_m(b_l^{ox}/b_l^{red}) = RT/(nF)\ \ln(b_l^{ox}/b_l^{red}) - RT/(nF)\ \ln(b_h^{ox}/b_h^{red});$$

$$E_m(b_h^{ox}/b_h^{red}) - E_m(b_l^{ox}/b_l^{red}) = RT/(nF)\ \ln(b_h^{red}\cdot b_l^{ox}/(b_h^{ox}\cdot b_l^{red})) \quad (9)$$

Since $Keq = k_f/k_r = b_h^{red}\cdot b_l^{ox}/(b_h^{ox}\cdot b_l^{red})$, the expression under logarithm in (9) equals to k_f/k_r:

$$E_m(b_h^{ox}/b_h^{red}) - E_m(b_l^{ox}/b_l^{red}) = RT/(nF)\ \ln(k_f/k_r)$$

$$k_f/k_r = \exp((nF/RT)\cdot(E_m(b_h^{ox}/b_h^{red}) - E_m(b_l^{ox}/b_l^{red})))$$

Parameters, their values and references:

b_l^{ox}/b_l^{red}	-58	Em, mV	Covian et al, 2007
b_l^{ox}/b_l^{red}	-142	Em, mV	Leguijt et al, 1993
b_h^{ox}/b_h^{red}	61	Em, mV	Covian et al, 2007
b_h^{ox}/b_h^{red}	116	Em, mV	Leguijt et al, 1993
ΔEm	119	mV	
$Keq=kf/kr=(b_h^{red}\cdot b_l^{ox})/(b_h^{ox}\cdot b_l^{red})$			
k_f/k_r	1.04E+002		

Reaction 4. Transport of first electron from b_h to Q on the n-side: $Q + b_h^{red} \leftrightarrow Q^- + b_h^{ox}$

At equilibrium the redox potentials for (b_h^{ox}/b_h^{red}) and (Q/Q^-) are equal:

$$E_m(b_h^{ox}/b_h^{red}) + RT/(nF)\ \ln(b_h^{ox}/b_h^{red}) = E_m(Q/Q^-) + RT/(nF)\ \ln(Q/Q^-); \qquad (10)$$

After simple rearrangement of the terms in (10):

$$E_m(Q/Q^-) - E_m(b_h^{ox}/b_h^{red}) = RT/(nF)\ \ln(b_h^{ox}/b_h^{red}) - RT/(nF)\ \ln(Q/Q^-);$$

$$E_m(Q/Q^-) - E_m(b_h^{ox}/b_h^{red}) = RT/(nF)\ \ln(b_h^{ox}\cdot Q^-/(b_h^{red}\cdot Q)) \qquad (11)$$

Since $Keq=k_f/k_r=b_h^{ox}\cdot Q^-/(b_h^{red}\cdot Q)$, the expression under logarithm in (11) equals to k_f/k_r:

$$E_m(Q/Q^-) - E_m(b_h^{ox}/b_h^{red}) = RT/(nF)\ \ln(k_f/k_r)$$

$$k_f/k_r = \exp((nF/RT)\cdot(E_m(Q/Q^-) - E_m(b_h^{ox}/b_h^{red})))$$

Parameters, their values and references, for the first electron:

b_h^{ox}/b_h^{red}	116	Em, mV	Leguijt et al, 1993
b_h^{ox}/b_h^{red}	61	Em, mV	Covian et al, 2007
Q/Q- at Qi	90	Em, mV	Covian et al, 2007
Q/Q- at Qi	73.1	Em, mV	Covian et al, 2007
Q/Q- at Qi	45	Em, mV	Rich PR, 1984
ΔEm	29	mV	
$Keq=kf/kr=(b_h^{ox}\cdot Q^-)/(b_h^{red}\cdot Q)$			
k_f/k_r	3.1001128	mV	

Reaction 5. Transport of second electron from b_h to Q^- on the n-side:

$$Q^- + b_h^{red} + 2H^+_n \leftrightarrow QH_2 + b_h^{ox}$$

At equilibrium the redox potentials for (b_h^{ox}/b_h^{red}) and (Q^-/QH_2) are equal:

$E_m(b_h^{ox}/b_h^{red}) + RT/(nF)\ ln(b_h^{ox}/b_h^{red}) = E_m(Q^-/QH_2) + RT/(nF)\ ln(Q^-/QH_2);$ (10)

After simple rearrangement of the terms in (10):

$E_m(Q^-/QH_2) – E_m(b_h^{ox}/b_h^{red}) = RT/(nF)\ ln(b_h^{ox}/b_h^{red}) – RT/(nF)\ ln(Q^-/QH_2);$

$E_m(Q^-/QH_2) – E_m(b_h^{ox}/b_h^{red}) = RT/(nF)\ ln(b_h^{ox}\cdot QH_2/(b_h^{red}\cdot Q^-))$ (11)

Since $Keq=k_f/k_r = b_h^{ox}\cdot QH_2/(H_2\cdot b_h^{red}\cdot Q^-)$, the expression under logarithm in (11) equals to $k_f\cdot H_2/k_r$:

$E_m(Q^-/QH_2) – E_m(b_h^{ox}/b_h^{red}) = RT/(nF)\ ln(k_f\cdot H_2/k_r)$

$k_f\cdot H_2/k_r = exp((nF/RT)\cdot(E_m(Q^-/QH_2) – E_m(b_h^{ox}/b_h^{red})))$

Parameters, their values and references:

b_h^{ox}/b_h^{red}	116	Em, mV	Leguijt et al, 1993
b_h^{ox}/b_h^{red}	61	Em, mV	Covian et al, 2007
Q^-/QH_2 at Qi	16.5	Em, mV	Covian et al, 2007
Q^-/QH_2 at Qi	150	Em, mV	Rich PR, 1984
ΔEm	0	mV	
$Keq=kf/kr =(b_h^{ox}\cdot QH_2)/(b_h^{red}\cdot Q^-\cdot H^2)$			
$k_f\cdot H^2/k_r$	1		

Model verification by simulation of the effects of ADP on respiration rate and ROS production

It is well known that when mitochondria are incubated with succinate in state 4 of respiration, addition of ADP essentially decreases ROS production (see e.g. Loschen et al, 1971; Korshunov et al 1997; Votyakova and Reynolds, 2001). Model simulation shown in **Figure S2a** explains this phenomenon. Mitochondria incubated with succinate in state 4 of respiration (without ADP) are characterized by high value of transmembrane potential. Simulations of electron transport for ΔΨ=200 mV and various succinate supply starting from oxidized and reduced initial conditions of respiratory chain revealed the area of bistability confined between blue and orange curves. Normally, mitochondria incubated with succinate without ADP are in high ROS producing mode (part of orange curve at succinate > 0.8). Addition of ADP induces ATP synthesis using the energy of $\Delta\mu H^+$, which signifies a decrease of transmembrane potential. Similar simulations for ΔΨ=150 mV showed that the area of bistability is shifted to the right and the same substrate supply of 0.8-0.9 now corresponds to a single steady state

with low semiquinone content (green curve). This means that if initially mitochondria are in state 4 in high ROS producing mode, addition of ADP would switch them to a low ROS producing mode as is observed in experiments.

According to the data of Panov and Scarpa (1996) respiration rate of rat liver mitochondria in state 4 is ~0.5 ng atom O/mg of protein/min. This electron flow in state 4 is equilibrated by proton leak, which depends on transmembrane potential, the higher potential, the higher leak. The potential restricts also the electron flow and the equilibrium between electron flow and leak is reached at the value of transmembrane potential of ~200 mV. After the addition of ADP the value of $\Delta\mu H^{+}$ decreases and this induces increase of respiration rate to the value of ~1.5 ng atom O/mg of protein/min.

Our model also describes the change in electron flow through the respiratory chain in response to the addition of ADP. The electron flows, which correspond to simulations shown in Figure S2a, are depicted in **Figure S2b**. High ROS producing mode at state 4 corresponds to the orange curve and it is around 0.4 ng atom of O/mg/s at succinate supply around 0.8-0.9. Addition of ADP signifies shift to the green curve (which at succinate 0.8-0.9 coincides with yellow curve) with the increase of electron flow up to 1.4 ng atom of O/mg/s.

It should be noted that the main results presented here and in the main text should be considered as a qualitative explanation of the observed phenomena of ROS production and flux change under various conditions based on the revealed bistable behavior of respiratory chain. In particular, it is important that the response to ADP application, such as decrease of ROS production and increase of flux could be explained in terms of bistability. However, the region of bistability could in reality be wider or more narrow; this is defined by specific values of parameters which in each particular case could differ from those accepted in the presented simulations despite the fact that we used the available data for their determination. Moreover, the contribution of the parts of intracellular oxidative system, which are not considered here, such as TCA cycle and complex I, could essentially affect the area of bistability. For instance, if the maximal activity of succinate dehydrogenase is restricted by 0.9 of our relative units, any variation of succinate concentration in fact will not allow the upper border of bistability region to be reached.

Figure S2b shows the restrictions of electron flow, which are internal with respect to Q-cycle mechanism. However, the present model does not consider the restrictions imposed by other processes. Specifically, blue curve in Figure S2b shows that in low ROS producing mode in state 4 at 0.8-0.9 of relative succinate supply electron flux could reach almost the same values as in state 3. However, such

high values could not be reachable because the regulation of TCA cycle will restrict succinate dehydrogenase activity at high levels of NADH.

The steady state properties of the system presented in Figures S2a and S2b allow to explain the switch from high to low ROS production induced by ADP and the switch back to high ROS production induced by hypoxia shown in Figure 3c of the main text. **Figure S2c** qualitatively simulates the dynamics of the observed shift between steady states. It shows the dynamics of transition from high to low ROS producing steady state induced by the addition of ADP, simulated as decrease of $\Delta\Psi$. This switch corresponds to the transition from orange to yellow curve shown in Figure S2a. When ADP is consumed ROS production rate changes in accordance with the low ROS producing state corresponding to the increased $\Delta\Psi$ (indicated by blue curve in Figure S2a). Hypoxia, simulated as decrease of rate constant of outflow from cytochrome c1, induced switch to high ROS producing state (indicated by orange curve in Figure S2a).

Sensitivity of the property of bistability to the model parameters

To investigate the property of bistability in mitochondrial respiratory chain operation, we checked how it is sensitive to the variation of model parameters. By definition, bistability is the existence of two different steady states of variables in a dynamic system, which corresponds to the same set of model parameters. The evolution of the system to one or another steady state is defined only by the initial values of variables. **Figures S3** and **S4** show that in a range of succinate supply, which provides electron flow within physiological range (as indicated in Figure S2b), there exist a region where the system comes to one of two different steady states of **p-side bound semiquinone content,** depending on whether the system starts from **oxidized** or **reduced** initial state. This region of bistability is sensitive to the variation in model parameters. Blue and orange lines indicate the bistable region, which corresponds to the indicated above set of parameters. Yellow and green lines illustrate how this region changes in response to the change of a parameter as indicated in the figures.

Ten-fold increase of k_f for transport of the first electron from QH_2 bound on the p-side to Fe^{3+} of Rieske protein (reaction 0 in the list above) practically did not change the bistability area as **Figure S3a** shows.

The change of ΔE_m for this reaction from -140 to 0 mV also insignificantly changed the bistability region.

For the reaction 1 ten-fold increase of k_f also practically did not change the bistability region, similarly, the increase of ΔE_m from –80 to 0 had little effect.

The change of ΔE_m from 0 to 50 mV for the transport of second electron from Q_p to b_l (reaction 2 in the list above) results in the decrease of the bistability area as **Figure S3b** shows.

Ten-fold increase of k_f for electron transport from b_l to b_h (reaction 3) shifts the bistability area towards higher succinate concentrations as **Figure S3c** shows.

Increase of ΔE_m in the same reaction from -250 mV to 0 shrinks this area as **Figure S3d** shows.

Five-fold increase of k_f for transport of first electron from b_h to Q on the n-side (reaction 4) from 83 to 400 s-1, shifts the bistability area as **Figure S3e** shows.

Decrease of ΔE_m in the same reaction to -100 mV expands this area as **Figure S3f** shows.

The bistability region is the most sensitive to the k_f of transport of the second electron from b_h to Q^- on the n-side (reaction 5). Two-fold increase of this parameter essentially expands the bistability region as **Figure S4a** shows. The decrease of ΔE_m for this reaction from 50 to 0 mV did not significantly change the region of bistability.

The ten-fold increase of k_f for ubiquinol binding to the complex III at the cytosolic side of the inner mitochondrial membrane shifts the bistability region as **Figure S4b** shows. The increase of K_d for reaction 6 to 1.2 nmol/mg.prot did not significantly change the region of bistability.

Three-fold increase of k_f for ubiquinone binding to the complex III at the matrix side of the inner mitochondrial membrane shrunk and shifted the bistability area as **Figure S4c** shows.

Decrease of K_d shifted bistability area to the right as **Figure S4d** shows.

k_f for ubiquinone binding to the complex III at the cytosolic side of the inner mitochondrial membrane increased three folds without essential changes in bistability region as **Figure S4e** shows.

Change of K_d for this reaction and the parameters of ubiquinol dissociation and binding to the complex III at the matrix side of the inner mitochondrial membrane (reaction 9) changed the area of bistability insignificantly.

The increase of the reaction rate constant for Q_o bound semiquinone interaction with molecular oxygen (ROS production) did not change the steady states when the system initially in low ROS producing mode, but facilitated the return lo low ROS production when initially it is in high ROS producing mode as **Figure S4f** shows.

Thus, the above analysis of sensitivity of the bistability to the variation of model parameters have shown that the property of bistability is robust, it persists over a large region of the variation in parameters in the physiolgical range of respiration rates, according to the conparison with the experimental data (Panov et al, 2005), discussed above. The bistability region is the most sensitive to

the k_f of transport of second electron from b_h to Q^- on the n-side (reaction 5). Increase of this parameter allows the essential expansion of the region of bistability. This reaction depends on proton concentration. In the simulation above, pH was set to 7.0, but acidification would expand the bistability area.

Redistribution of redox states, which define the transition of complex III from low to high ROS producing mode.

Figure S5 illustrates the distribution of redox states at a steady state low ROS producing mode, transient intermediate state and at a steady state high ROS producing mode. After transition from low to high ROS production all redox states essentially redistributed.

Additional experimental data, which were not present in the main text.

The data shown in Figure 3a of the main text show that if ADP is initially present in the medium it switches the mitochondrial respiratory chain to low ROS production, which persisted after total conversion of ADP into ATP. The fact that ROS production decreased in the presence of ADP was observed previously (Loschen et al. 1971; Boveris et al. 1972; Boveris and Chance 1973; Korshunov et al., 1997; Votyakova and Reynolds, 2001) and explained as an effect of decreased transmembrane potential, which affects reverse electron flow. Here we present evidence that low rate of ROS production persists after ADP is consumed and transmembrane potential is restored. This evidence indicates that ADP plays a role of a trigger, switching the electron transport from high to low ROS producing steady states persisting under the same microenvironmental conditions. Moreover, if initially ADP was absent in the incubation medium and added later, when high ROS production was already registered, this addition also triggers mitochondrial respiration to lower ROS production persisting after ADP consumption, as **Figure S6a** of this Supporting Information shows.

ADP added to respiring mitochondria is converted into ATP. **Figure S6b** shows that ATP itself does not affect ROS production. Black curve in Figure S6b indicates that, if ATP has been added with oligomycin, ROS production has not changed. Oligomycin was added to avoid the stimulation of electron transport, which could be induced by ADP present in the preparation of ATP. If ATP is added without oligomycin, short decrease of transmembrane potential, induced by the phosphorylation of ADP, is observed. During this phosphorylation, ROS production decreased and then, although increased, did not reach the initial level. Thus, even short stimulation of electron transport by low amount of ADP was sufficient to switch ROS production in a part of mitochondria. Addition of oligomycin after ATP (green line) did not restore the rate of ROS production. Apparently the degree of

ROS rate restoration after completion of ADP phosphorylation depends upon the length of state 3: the longer mitochondria persisted in state 3, the lower the consequent rate of ROS production.

References

1. Boveris A. and Chance B. (1973) The mitochondrial generation of hydrogen peroxide: general properties and effect of hyperbaric oxygen. *Biochem. J.* **134**, 707-716.
2. Boveris A., Oshino N. and Chance B. (1972) The cellular production of hydrogen peroxide. *Biochem. J.* **128**, 617-630.
3. Covian R, Zwicker K, Rotsaert FA, Trumpower BL. (2007) Asymmetric and redox-specific binding of quinone and quinol at center N of the dimeric yeast cytochrome bc1 complex. Consequences for semiquinone stabilization. *J Biol Chem.* **282**, 24198-24208.
4. Demin, O.V., Kholodenko, B.N., & Skulachev, V.P. (1998) A model of O_2^- generation in the complex III of the electron transport chain . *Mol. Cell. Biochem*. **184**, 21–33 .
5. Ding H, Moser CC, Robertson DE, Tokito MK, Daldal F, Dutton PL. (1995) Ubiquinone pair in the Q_o site central to the primary energy conversion reactions of cytochrome bc1 complex. *Biochemistry* 34, 15979-15996.
6. Dutton PL, Moser CC, Sled VD, Daldal F, Ohnishi T. (1998) A reductant-induced oxidation mechanism for complex I. *Biochim Biophys Acta*. **1364,** 245-257.
7. Gennis R.B. (1972) Biomembranes: Molecular Structure and Functions, NewYork, 1989;
8. Green, D.E., Wharton, D.C. (1963) Stoichiometry of the fixed oxidation-reduction components of the electron transfer chain of beef heart mitochondria. *Biochem. Z.* **336**, 335–346 .
9. Hansford, R. G., Hogue, B. A., and Mildaziene, V. (1997) Dependence of H2O2 formation by rat heart mitochondria on substrate availability and donor age. *J Bioenerg Biomembr* **29**, 89-95.
10. Korshunov, S. S., Skulachev, V. P., and Starkov, A. A. (1997) High protonic potential actuates a mechanism of production of reactive oxygen species in mitochondria. *FEBS Lett* **416**, 15-18.
11. Leguijt T, Engels PW, Crielaard W, Albracht SP, Hellingwerf KJ. (1993) Abundance, subunit composition, redox properties, and catalytic activity of the cytochrome bc1 complex from alkaliphilic and halophilic, photosynthetic members of the family Ectothiorhodospiraceae. *J Bacteriol.* **175**, 1629-1636.
12. Loschen G., Flohe L. and Chance B. (1971) Respiratory chain linked H2O2 production in pigeon heart mitochondria. *FEBS Lett*. **18**, 261-264.
13. Mulkidjanian AY (2005) Ubiquinol oxidation in the cytochrome bc 1 complex: Reaction mechanism and prevention of short-circuiting. *Biochim Biophys Acta* **1709,** 5 – 34.
14. Ohnishi ST, Ohnishi T, Muranaka S, Fujita H, Kimura H, Uemura K, Yoshida K,Utsumi K (2005) A Possible Site of Superoxide Generation in the Complex I Segment of Rat Heart Mitochondria. *J. Bioenerg. Biomembr.* **37**, 1-15.
15. Ohnishi T (1998) Iron-sulfur clusters/semiquinones in complex I. *Biochim Biophys Acta*. **1364**, 186-206.
16. Ohnishi T, Salerno JC. (2005) Conformation-driven and semiquinone-gated proton-pump mechanism in the NADH-ubiquinone oxidoreductase (complex I). *FEBS Lett*. **579**, 4555-61.

17. Ohnishi T, Johnson JE Jr, Yano T, Lobrutto R, Widger WR. (2005) Thermodynamic and EPR studies of slowly relaxing ubisemiquinone species in the isolated bovine heart complex I. *FEBS Lett*. **579**, 500-506.

18. Panov A, Scarpa A. (1996) Mg2+ control of respiration in isolated rat liver mitochondria. *Biochemistry*. **35,** 12849-12856.

19. Panov A, Dikalov S, Shalbuyeva N, Taylor G, Sherer T, Greenamyre JT. (2005) Rotenone model of Parkinson disease: multiple brain mitochondria dysfunctions after short term systemic rotenone intoxication. *J. Biol. Chem*. 280, 42026-42035.

20. Reynolds IA, Johnson EA, Tanford C (1985) Incorporation of membrane potential into theoretical analysis of electrogenic ion pumps. *Proc Natl Acad Sci USA* **82,** 6869–6873.

21. Salerno, J.C., Ohnishi, T. (1980) Studies on the stabilized ubisemiquinone species in the succinate cytochrome c reductase segment of the intact mitochondrial membrane. *Biochem. J.* **192**, 769-781 .

22. Schumacker, P.T. (2006) Reactive oxygen species in cancer cells: live by the sword, die by the sword. *Cancer Cell.* **10**, 175-176.

23. Sled VD, Rudnitzky NI, Hatefi Y, Ohnishi T. (1994) Thermodynamic analysis of flavin in mitochondrial NADH:ubiquinone oxidoreductase (complex I). *Biochemistry*. **33,** 10069-10075.

24. Trumpower BL. (1981) Function of the iron-sulfur protein of the cytochrome b-c1 segment in electron-transfer and energy-conserving reactions of the mitochondrial respiratory chain. *Biochim Biophys Acta.* **639**, 129-155.

25. Trumpower BL. (1981a) New concepts on the role of ubiquinone in the mitochondrial respiratory chain. *J Bioenerg Biomembr*. **13**, 1-24.

26. Vinogradov AD. (1998) Catalytic properties of the mitochondrial NADH-ubiquinone oxidoreductase (complex I) and the pseudo-reversible active/inactive enzyme transition. *Biochim Biophys Acta.* **1364**, 169-185.

27. Votyakova, T.V., Reynolds I.J (2001) DCm-Dependent and -independent production of reactive oxygenspecies by rat brain mitochondria. *Journal of Neurochemistr*y **79**, 266-277.

28. Yu, C.-A. *et al.* (1999) Structure and reaction mechanisms of multifunctional mitochondrial cytochrome bc1 complex. *BioFactors* **9**, 103–109.

29. Zhang, H., Gajate, C., Yu, L.P., Fang, Y.X., Mollinedo, F. (2007) Mitochondrial-derived ROS in edelfosine-induced apoptosis in yeasts and tumor cells. *Acta Pharmacol. Sin.* **28**, 888-894.
