# Supplementary figures and images for: Bistability of Mitochondrial Respiration Underlies Paradoxical Reactive Oxygen Species Generation Induced by Anoxia
Source: PLoS Comput Biol. 2009 Dec 24;5(12):e1000619. doi: 10.1371/journal.pcbi.1000619 (PMC2789320; doi:10.1371/journal.pcbi.1000619)

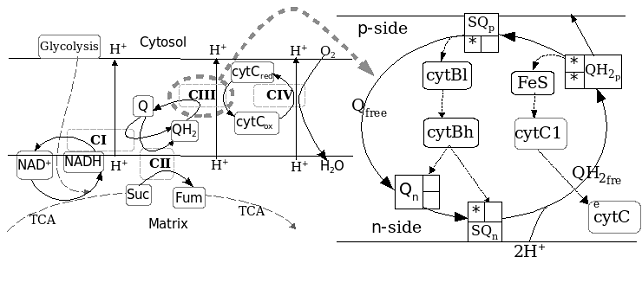

Supplement: Figure S1 — Scheme of the processes in mitochondrial electron transport chain and their coupling with central energetic metabolism. Respiratory complexes (CI–CIV) deliver electrons from NADH oxidizing it to NAD+ to oxygen reducing it to H2O. This delivery is subdivided into many steps, some of which are coupled with proton transport from matrix to cytosol. Redox reactions in electron transport chain and proton translocation are fed by glycolysis and TCA cycle, which provide NADH, complex 1 substrate, and succinate, complex II substrate. Ubiquinone/ubiquinol (Q/QH2) cycle plays essential role in proton transport and communication between CI–CIII. The scheme of electron transport coupled with proton translocation in the complex III (Q-cycle mechanism) is shown in more detail in the right panel. Oxidizes ubiquinol reducing cytochrome C and releasing two protons to the cytosolic side (positive or p-side; this is reflected in the index of released H+). In addition it translocates two protons from matrix (negative or n-side) to cytosol. Ubiquinol (QH2) delivers its first electron to Fe(3+), releasing 2 protons at the p-side of the inner mitochondrial membrane and producing semiquinone (SQ) radical. Then the latter gives its unpaired electron to cytochrome bl and produced ubiquinone (Q) dissociates from the complex. Free Q is bound at the n-side, receives two electrons from cytochrome bh resulted from oxidation of two QH2 molecules, thus producing subsequently SQ and QH2, taking protons from n-side. Dissociation of the produced QH2 accomplishes a round of the cycle. (0.06 MB TIF) [file pcbi.1000619.s002.tif]

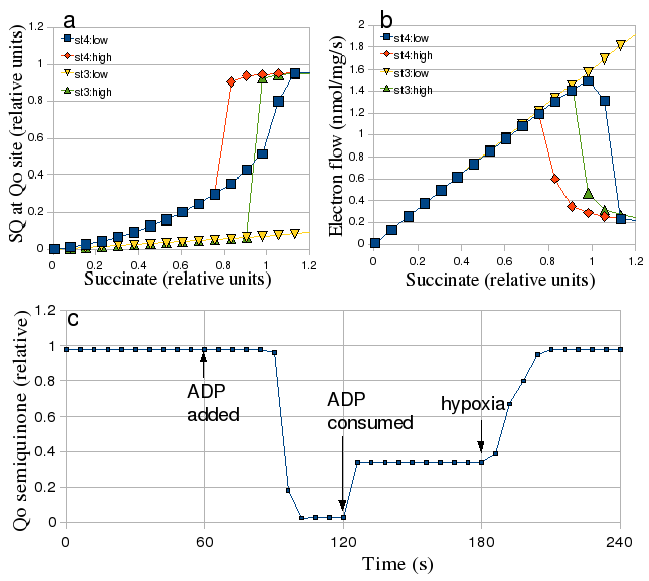

Supplement: Figure S2 — Validation of the model by qualitative simulation of the observed experimentally decrease of ROS production and increase of maximal flow rate induced by ADP, and increase of ROS production induced by hypoxia. (a) Decrease of steady state content of semiquinone bound at Qo site (ROS production rate) in conjunction with a decrease of transmembrane potential. Blue squares indicate the steady state levels of SQ bound at Qo site reached from initial states with high SQ levels at various relative succinate concentrations. Orange diamonds indicate the steady state SQ at Qo levels reached from initial states with low SQ levels. These two curves, which indicate bistable behavior of complex III operation, were obtained for transmembrane potential 200 mV that simulates state 4 of respiration. If transmembrane potential falls down to below 150 mV (as could be after a transition to state 3 induced by addition of ADP that stimulates ATP synthesis), the area of bistability is shifted to the right (as indicated by yellow and green triangles) and the same substrate supply corresponds now to low semiquinone content. Thus, if the system operates in high ROS producing mode at the relative succinate levels around 0.8–0.9, addition of ADP would switch it to the low ROS producing mode. (b) Increase of electron flow through the respiratory chain in conjunction with a decrease of transmembrane potential. The labels of curves correspond to the same conditions as in Figure S2a. If the system persists in state 4 of respiration in a high ROS producing mode (orange diamonds), the addition of ADP (transition to state 3) would switch the electron flow to the values indicated by green triangles, and if the substrate supply is around 0.8–0.9, electron flow would increase from 0.4 to 1.4 ng.atom of O/mg/s. (c) Initially mitochondria incubated with succinate are in state 4 and in high ROS production state (orange curve in Figure S2a). The addition of ADP, simulated as decrease of transmembrane potenti [file pcbi.1000619.s003.tif]

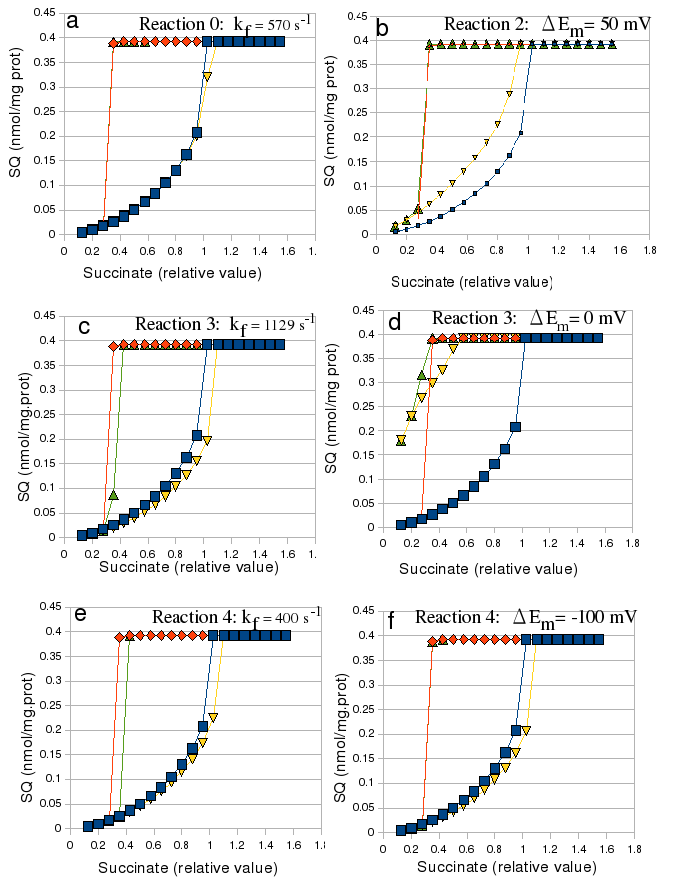

Supplement: Figure S3 — Analysis of sensitivity of the bistability region to the change of model parameters. The basic model parameters, which are listed above and in the main text. To show the sensitivity of bistability region the parameters were changed, which induced the change of area of bistability. Blue and orange lines indicate the bistable region, which corresponds to the indicated above set of parameters. Yellow and green lines illustrate how this region changes in response to the change of a parameter as indicated in the figures. (0.11 MB TIF) [file pcbi.1000619.s004.tif]

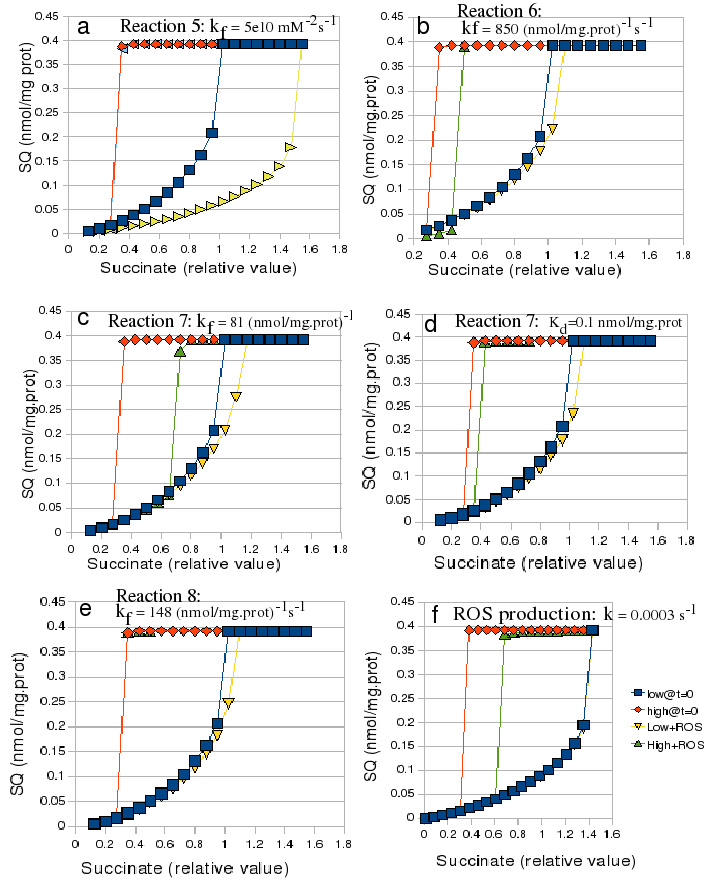

Supplement: Figure S4 — Analysis of sensitivity of the bistability region to the change of model parameters. Designations are the same as in Figure S3 (0.12 MB TIF) [file pcbi.1000619.s005.tif]

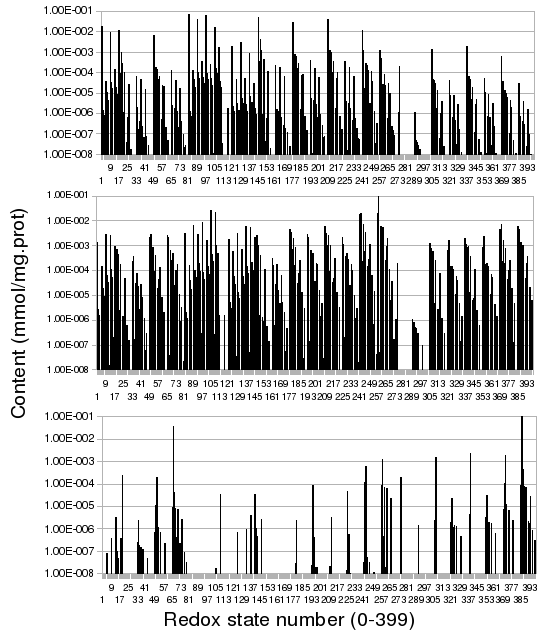

Supplement: Figure S5 — Distribution of redox states of complex III, which corresponds to low (upper panel), high (lower panel) ROS production states or intermediate non-steady state reached in 3 second after the switch of substrate supply which induced the transition from low to high ROS producing steady state (middle panel). The redox states are positioned as follows: the numbers 0–15 correspond to 16 states of bh-bl-c1-FeS, numbers 16–79 correspond to 64 states of bh-bl-c1-FeS-Q-Q, numbers 80–143 correspond to 64 states of Q-Q-bh-bl-c1-FeS, numbers 144–399 correspond to 256 states of Q-Q-bh-bl-c1-FeS-Q-Q. (0.08 MB TIF) [file pcbi.1000619.s006.tif]

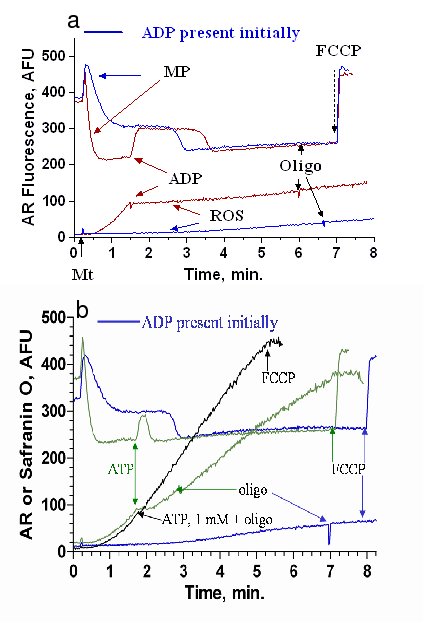

Supplement: Figure S6 — (a) Temporary stimulation by ADP switches the electron transport from high to low ROS production mode. Rat brain mitochondria were added to the incubation medium, with subsequent addition of 1 mM ADP (red curves), or with 1 mM of ADP present initially (blue curves). Without ADP, ROS production is much higher than in its presence, however, after ADP addition it decreases. During the transformation of ADP into ATP the membrane potential remains low but, when all added ADP is transformed, the potential increases (curves MP). However, after the completion of transformation, ROS production increased, but not to the original level. Since the produced ATP itself does not affect ROS production, this data indicate that ADP plays a role of trigger, which switches the respiratory chain from high to low ROS production. (b) ATP does not affect ROS production, but adventitious ADP in the ATP preparation was sufficient to switch the electron transport to low ROS producing mode. Blue curves are shown for comparison; they are taken from an experiment, similar to that shown in (a). Black curve shows that ATP added with oligomycin does not change ROS production. Green curves show that a presence of traces of ADP in the preparation of ATP switches a part of mitochondria to low ROS producing mode, and that the addition of oligomycin after ATP does not bring the ROS rate to the original level. (0.05 MB TIF) [file pcbi.1000619.s007.tif]
